# Suppression of seizure in childhood absence epilepsy using robust control of deep brain stimulation: a simulation study

**DOI:** 10.1038/s41598-023-27527-1

**Published:** 2023-01-10

**Authors:** Ehsan Rouhani, Ehsan Jafari, Amir Akhavan

**Affiliations:** 1 Department of Electrical and Computer Engineering, Isfahan University of Technology, Isfahan, 84156-83111 Iran; 2 CNRS UMR 5672, Ecole Normale Supérieure de Lyon, 46 allée d’Italie, 69007 Lyon, France

**Keywords:** Neurological models, Dynamical systems, Computational models, Disease model, Epilepsy, Epilepsy, Biomedical engineering

## Abstract

Deep brain stimulation (DBS) is a promising technique to relieve the symptoms in patients with intractable seizures. Although the DBS therapy for seizure suppression dates back more than 40 years, determining stimulation parameters is a significant challenge to the success of this technique. One solution to this challenge with application in a real DBS system is to design a closed-loop control system to regulate the stimulation intensity using computational models of epilepsy automatically. The main goal of the current study is to develop a robust control technique based on adaptive fuzzy terminal sliding mode control (AFTSMC) for eliminating the oscillatory spiking behavior in childhood absence epilepsy (CAE) dynamical model consisting of cortical, thalamic relay, and reticular nuclei neurons. To this end, the membrane voltage dynamics of the three coupled neurons are considered as a three-input three-output nonlinear state delay system. A fuzzy logic system is developed to estimate the unknown nonlinear dynamics of the current and delayed states of the model embedded in the control input. Chattering-free control input (continuous DBS pulses) without any singularity problem is the superiority of the proposed control method. To guarantee the bounded stability of the closed-loop system in a finite time, the upper bounds of the external disturbance and minimum estimation errors are updated online with adaptive laws without any offline tuning phase. Simulation results are provided to show the robustness of AFTSMC in the presence of uncertainty and external disturbances.

## Introduction

Epilepsy refers to a set of chronic neurological diseases typified by repetitive (at least two) spontaneous seizure attacks due to abnormal bursts of electrical activity in the brain that can bring about temporary brain dysfunction^1^. According to the regional emergence of excessive electrical activity in the brain, the International League Against Epilepsy (ILAE) classified seizures as focal (partial), generalized, and unknown^2,3^. Focal seizures are established in a local area of the brain. They can be further classified based on the patient's awareness during a seizure attack as aware (simple) and impaired awareness (complex). In generalized seizures, both hemispheres are involved at the onset of the seizure, and awareness is impaired in most cases. The regional onset is unspecified in unknown seizures, but other manifestations are known^3^. These classes can also be subdivided into the motor or non-motor subsets regarding movement manifestations^2–4^. Childhood absence epilepsy (CAE) is a subset of genetic/idiopathic generalized epilepsy characterized by frequent daily absence (non-motor) seizures in children between 2 to 13 years old. The typical features of CAE include sudden cessation of activity and awareness (e.g., staring blankly), short time duration episodes (approximately 9 s), and 2.5–3.5 Hz bilateral, synchronous spike-and-wave discharges (SWDs) in the recorded electroencephalogram (EEG) signal during seizure episodes in the brain^5,6^. Elucidating the pathogenesis of CAE, as well as developing efficacious remedies, are the fundamental challenges that should be tackled by researchers.

Various studies on patients with absence seizures and animal models acknowledged that the SWDs in electrophysiological recordings mainly originated from the aberrant neuronal interplay between the cerebral cortex and thalamus that constitutes the corticothalamic network. In fact, the reticular thalamic nucleus, thalamic relay nuclei, and cortical neurons form a reciprocal neuronal circuit demonstrating a significant contribution in the absence of seizure onset^7–13^. In accordance with experimental results, numerous computational corticothalamic models have been developed to discover the underlying mechanisms of transition from regular brain activity to SWDs^14–25^. To replicate the spontaneous SWDs in EEG recordings of rats with the absence of epilepsy, Suffczynski et al.^21^ developed a network model of the corticothalamic circuit in which the thalamus model consisted of the thalamocortical relay cells and reticular nucleus, and the cortex role imitated by the excitatory pyramidal cell population and inhibition interneuron population. Using these mutually interconnected neuronal populations, SWDs can be rendered by dynamical bifurcation in a bistable system. Later Rodrigues et al.^22,23^ recruited a mean-field model of the human cortico-thalamic circuit to present the transition from the inter-ictal phase to SWD. Marten et al.^24^ presented a modified mean-field model of human EEG activity that could explain not only the classical SWDs dynamics but also more dynamical phenomena, e.g., polyspike complexes. Destexhe^7,8^ developed a conductance-based neuronal model of the thalamocortical circuit and emphasized the pivotal role of corticothalamic feedback and GABA receptors in hyper-synchronized oscillations and SWDs patterns. Motivated by this work, a 3-neuron pattern of CAE was formulated^25^. According to this model, long corticothalamic loop delay and low GABA_A_ activity of thalamic relay cells are associated with CAE. Despite GABA_A_ deficiency, it was demonstrated that axonal myelination, which occurs by reaching adulthood, might shorten the corticothalamic loop delay and lead to outgrowing seizures.

CAE might negatively impact school-aged children's social behavior, self-confidence, and education, accentuating the necessity of prompt and efficient treatment^26,27^. Although antiepileptic drugs are the most common prescription for CAE patients, they are ineffective in intractable cases, and long-term usage might cause side effects, e.g., liver disorders^28^. Neurosurgical resection, which is the removal of brain tissues contributing to epilepsy, is risky due to the generalized nature of the absence of epilepsy and irreversible consequences^29–31^.

Deep brain stimulation (DBS), the application of electrical pulses to specific brain regions using implanted electrodes, is often employed to treat movement disorders such as Parkinson’s disease (PD)^32,33^ as well as to relieve and control the symptoms in patients with intractable seizures^34^. Although the DBS therapy for seizure suppression dates back more than 40 years^35,36^, the outcomes left much to be desired. A thorough understanding of the underlying mechanism by which DBS terminates the seizure is of great importance. Concerning the cardinal role of the thalamus in seizure development and inhibition, the anterior and centromedian nuclei of the thalamus have been targeted to suppress refractory epilepsy^37^. Despite the promising results, the mechanism of action of DBS is not thoroughly clear. From the cellular perspective, DBS can regulate the activity of GABAergic neurons located in the reticular thalamic nucleus to produce inhibitory postsynaptic potentials and induce synaptic inhibition of excitatory corticothalamic relay neurons^38^. It has been demonstrated that the strength and duration of absence epilepsy are weakened by amplifying the GABAergic inhibitory power^39^.

Determining stimulation parameters such as amplitude, pulse width, and frequency is a significant challenge to the success of DBS technique. Open-loop control^40–46^ and closed-loop control^47–54^ have been the two main approaches to address this challenge. However, the open loop control approach strictly depends on the neurologist’s experience and provides constant pre-determined stimulation on either a continuous^43,44^ or periodic^42^ basis, which gives rise to excessive battery usage and, eventually, battery replacement surgery^55,56^. Moreover, persistent brain electrical stimulation without considering the current state of the disease interrupts the patient’s regular activity and might cause involuntary movements^57^. On the contrary, a closed-loop control system monitors the disease biomarkers and automatically provides real-time variable intensity stimulations, taking into account the onset and strength of the attacks^52^. Motivated by these benefits, various closed-loop control DBS schemes have been designed to treat neurological and neuropsychiatric disorders^58–62^. In^58^, a data-driven linear state-space model in which the parameters were learned by a novel input waveform was developed to build a dynamic input–output framework for neural activities. Furthermore, a real-time closed-loop simulation testbed to control the mood in depression was implemented. In^59^, a population of 100 thalamic neurons model was simulated to evaluate the effect of extracellular electrical stimulation on the generated local field potentials (LFPs) in tremor conditions. An autoregressive with exogenous input (ARX) model was employed to model the relationship between the stimulus current and LFPs. To restore the power spectral profiles of tremor-free conditions, an adaptive minimum variance controller was adopted, which regulates the stimulation intensity and thereby corrects the abnormal patterns of neuronal activity. Ehrens et al.^60^ used a fragile stochastic neuronal network model, which entails seizure and non-seizure modes regarding the synaptic weights between neurons. A hidden Markov model (HMM) was established to detect the seizure occurrences (unstable modes) followed by a state feedback gain to suppress the seizure attacks. To suppress the motor symptoms of PD, Su et al.^61^ proposed a DBS framework based on proportional-integral (PI) control to track the dynamic beta oscillatory activity presented in reference signals during voluntary movement. A linear controlled auto-regressive model was employed to represent the relationship between stimulation frequency and beta band power and then was coupled with Routh-Hurwitz stability analysis to tune the coefficients of the PI controller. In^63^, a Radial basis function neural network (RBFNN) was used in a supervisory control algorithm as an inverse model of the computational model of PD to track the desired dynamic beta power. To track the model uncertainty and provide robustness to noise and disturbance in control of brain states using DBS, Fang et al.^64^ proposed an adaptive robust controller to cancel uncertainties included in a state-space brain network model. Monte Carlo simulations were performed to validate the suggested algorithm.

Myriad closed-loop control systems were developed in recent years to suppress the epileptic seizures generated by computational models of epilepsy^47–51,54^. Wang et al.^47^ proposed a PI controller to control the high-amplitude seizure activity simulated by a neural mass model (NMM). Researchers in^50^, investigated an NMM representing the epileptic activities of the brain as a black box and established an auto-regressive moving-average Volterra model to approximate the black box input (stimulation)-output (EEG) relationship. Then, a model predictive controller was developed to remove the epileptic waves. Zhang et al.^54^ designed two closed-loop PI controllers to eliminate SWDs by automatically regulating the amplitude and frequency of DBS. To this end, a basal ganglia-corticothalamic model of absence epilepsy was approximated using the linear controlled auto-regressive model and recursive least square methods. The coefficients of the PI controllers were determined by the Routh-Hurwitz stability criterion. Despite the promising results exhibited by the mentioned control methods, the unmodeled dynamics and parameter uncertainties due to the highly nonlinear and time-varying nature of the neurological systems, external disturbance rejection, and stable function of the controller in real applications have not been entirely addressed.

Apart from simulation studies, a handful of clinical DBS systems to suppress epilepsy have been developed in the last two decades. Responsive Neurostimulation System (RNS, NeuroPace) is the first FDA-approved commercially available closed-loop device that provides responsive brain stimulation with respect to the electrocorticogram (ECoG) recordings through one or two depth and/or subdural cortical strip leads that are implanted at the seizure focus of focal onset seizure patients. This device demonstrated favorable outcomes on 11 out of 27 patients with epilepsy^65–67^. In^68^, an implantable concurrent sensing and stimulation DBS device that relies on LFP and ECoG sensors was developed and validated in an ovine model of epilepsy. A support vector machine (SVM) classification algorithm was recruited to separate the biomarkers from the stimulation artifact. DiLorenzo et al.^69^ devised a seizure advisory system in connection with an implantable 16-channels intracranial monitoring to predict seizures. The preclinical tests were performed in a canine model and then implantation of 15 patients to evaluate the method.

Sliding mode control (SMC) is a powerful nonlinear robust control strategy featuring outstanding performance to compensate for the parameter uncertainty and bounded external disturbances in highly nonlinear systems^70,71^. To achieve seizure abatement, a classical SMC was integrated with an RBFNN approximator to propel the EEG waves recorded from cortical areas with undesired dynamics to track the normal background waves^49^. In this work, the DBS-corticothalamic system was formulated to a canonical structure, and the system’s dynamics were approximated by RBFNN, while disturbance rejection and model uncertainties were handled by the classical SMC. The main disadvantage of the proposed controller is the asymptotic stability of the SMC due to the linear structure of the sliding surface. To tackle the stability problem, terminal sliding mode (TSM) control that contains fractional order of sliding surfaces was developed to ensure the finite-time convergence of the system trajectories to the origin^70,72^. However, TSM cannot address the chattering phenomena^70^ of the control input, which endangers the system’s safety in real applications. Moreover, it suffers from the singularity problem that may lead to infinite control input to guarantee the ideal TSM motion^73–75^. Qian et al.^51^ presented a finite-time fractional order SMC in combination with RBFNN to suppress epilepsy seizures in a well-established thalamocortical NMM. To design and stability analysis of the closed-loop system, an appropriate coordinate transformation was introduced to represent a regular form of the NMM. The main bottleneck of the work is that to transform the original NMM to the regular form and design the controller signal, prior knowledge of the NMM dynamics is necessary. To overcome the aforementioned problems (asymptotic stability, chattering, and singularity of the controller and the knowledge of the plant dynamics), in this paper, a robust control technique based on adaptive fuzzy TSM control (AFTSMC) is developed for control of the oscillatory spiking pattern of the three-neuron pattern of CAE whereby the controller is not dependent on any knowledge of the dynamics of the system to be controlled. The main contributions of this work are as follows:

- To implement the proposed MIMO control algorithm, the dynamics of the membrane voltage of three-neuron CAE motif^8,25^ should be represented and modeled as a three-input three-output nonlinear state delay model, and three continuous DBS pulses (control input) are designed to force the CAE pattern to track the normal state. This representation is based on the decomposition of the nonlinear dynamics of the current and delayed states of the CAE system^8,25^. Indeed, the continuous DBS pulses are automatically generated based on the MIMO model of the CAE motif. To achieve this, the unknown nonlinear dynamics of the MIMO model are approximated with the fuzzy logic system (FLS).
- A fast TSM-type reaching law is embedded in the continuous nonsingular control input to accelerate the speed of the motion to the sliding surface far away from the surface in finite time. The superiority of the continuous control law is that it’s chattering-free.
- On the sliding surface, the fractional-integral structure of the surface guaranteed the convergence of the tracking error to the neighborhood of zero in finite time.
- The nonlinear dynamics of the current and delayed states of the model embedded in the control input are approximated with the FLS, and adaptive laws based on the terminal gradient descent algorithm are developed for online updating the weights of the estimator.
- To guarantee the bounded stability of the closed-loop system, the upper bounds of the external disturbance and minimum estimation errors are updated online using terminal-based adaptive laws without any offline tuning phase.

## Model and control problem

### Model

**Figure 1:**
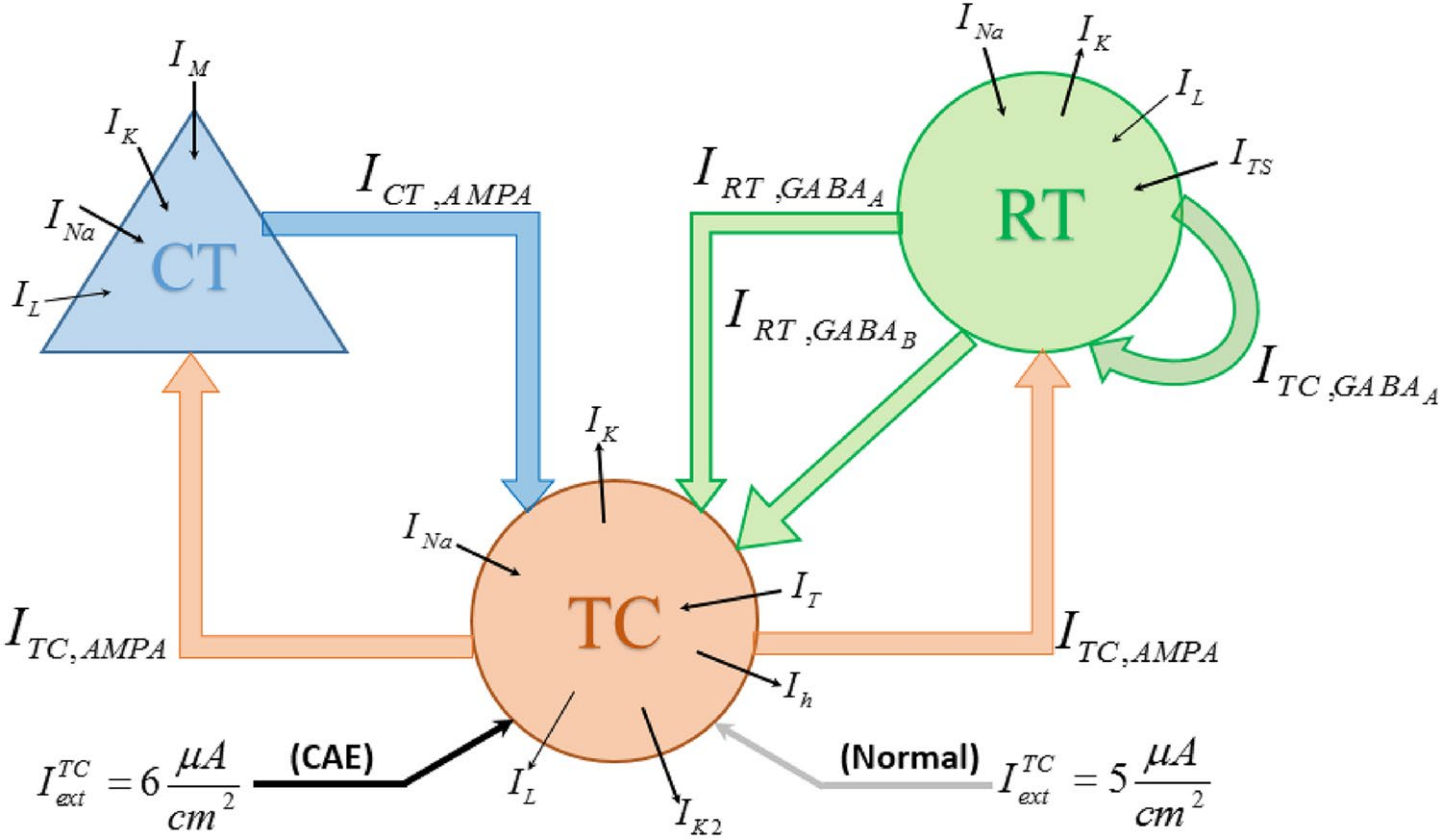
The topology of the three coupled TC, CT, and RT nuclei neurons.

CAE is a 2.5–3.5 Hz bilateral, synchronous sudden spike followed by the wave discharges (SWDs) observed in the EEG pattern of the children during seizure episodes. SWD patterns with similar characteristics in human subjects were also reported in the experimental works conducted on animal homologs. Electrophysiological recordings of the spike-and-wave seizures in animal models showed the aberrant neuronal interplay between the cerebral cortex and the two main thalamic cells involved thalamocortical and the inhibitory neurons of the thalamic reticular nucleus. These experiments suggested that the connections of the GABA_A_ and GABA_B_ receptors can play an important role in the activation of spike-and-wave seizures. It has been proven that the seizure appeared in the mouse homolog by reduction of the conductance of GABA_A_ receptors. Furthermore, both the thalamus and cortex are essential to the absence of seizures. As each cell type of the thalamus and cortex neurons contain the voltage-dependent currents necessary to define their properties, the dynamics of the electrical activity of the cortical (CT), thalamic relay (TC), and reticular (RT) nuclei neurons are represented as a conductance-based Hodgkin-Huxley type neuron model. The topology of the three-coupled network is depicted in Fig. 1, and the dynamics of the membrane voltage of the neurons are modeled with the following nonlinear first-order differential equations^8,25^:

1 $$\begin{aligned} C_{TC} \frac{{dV_{TC} }}{dt} & = - I_{L} - I_{Na} - I_{K} - I_{T} - I_{h} - I_{K2} - I_{syn}^{TC} + I_{ext}^{TC} \hfill \\ C_{CT} \frac{{dV_{CT} }}{dt} & = - I_{L} - I_{Na} - I_{K} - I_{M} - I_{syn}^{CT} + I_{ext}^{CT} \hfill \\ C_{RT} \frac{{dV_{RT} }}{dt} & = - I_{L} - I_{Na} - I_{K} - I_{TS} - I_{syn}^{RT} + I_{ext}^{RT} , \hfill \\ \end{aligned}$$

where in (1), $C_{TC} = C_{CT} = C_{RT} = 1$ μF cm^−2^ is the capacitance of the membrane of CT, TC, and RT neurons and $V_{TC}$, $V_{CT}$, and $V_{RT}$ is the membrane voltage of the CT, TC, and RT neurons, respectively. The ionic membrane currents (μA cm^−2^) are denoted by $I_{L}$, a leak current, $I_{Na}$, a transient voltage-gated current of Na^+^ ions, $I_{K}$, a transient voltage-gated current of K^+^ ions, $I_{TS}$, a low-threshold current of Ca^++^ ions for RT neuron, $I_{M}$, a depolarization-activated current of K^+^ ions, $I_{T}$, a low-threshold current of Ca^++^ ions for TC neuron, $I_{h}$, a mixed Na^+^-K^+^ current activated by hyperpolarization, $I_{K2}$, a slow current of K^+^ ions, and, $I_{ext}^{TC}$, $I_{ext}^{CT}$, and $I_{ext}^{RT}$, external currents of TC, CT, and RT neurons, respectively due to the input from other brain regions. Without the coupling effect of synaptic currents, if the external current of the neurons is set at zero, the voltage of the three neurons is at rest, and for a large nonzero value of the external currents, the voltage of the neuron is denoted with periodic spiking activity. In the simulation, the parameters of the CT and RT external current are set at 0 μA cm^−2^ and $I_{TC}$ is assumed to be 5 or 6 μA cm^−2^. Detailed dynamics of the ionic membrane currents are provided in Appendix A of the Supplementary Materials.

The synaptic currents ($I_{syn}^{i} ,i \in \left\{ {TC,CT,RT} \right\}$) are in the following form:

2 $$\begin{aligned} I_{syn}^{TC} & = I_{AMPA}^{CT} + I_{{GABA_{A} }}^{RT} + I_{{GABA_{B} }}^{RT} \hfill \\ I_{syn}^{CT} & = I_{AMPA}^{TC} \hfill \\ I_{syn}^{RT} & = I_{{GABA_{A} }}^{RT} + I_{AMPA}^{TC} , \hfill \\ \end{aligned}$$

where the general form of AMPA, GABA_A_, and GABA_B_ currents with the value of their parameters are summarized in Table 1. In the current equations, the parameters *E* (mv) and $\overline{g}$ (mS cm^−2^) are the reversal potential, and maximal conductance, respectively, and *s* is the fraction of the channels opened with the presynaptic neuron. In the dynamic equation of *s*, the parameter *T* is the concentration of neurotransmitters released with the presynaptic neuron, and the parameters $\alpha$ (m/s m/M) and $\beta$ (m/s) are two constants. Two time delays $\tau_{1}$ and $\tau_{2}$ are included in the model of neurotransmitter concentration as the conduction delay for the corticothalamic and thalamocortical delays, respectively. In the simulation, the thalamocortical delay is set to 2.8 ms and the corticothalamic delay is varied between 2 and 10 ms based on the dynamics of normal and CAE states. In the model of the synaptic current from the RT neuron to the TC neuron due to the (GABA_B_), the parameter $r_{{GABA_{B} }}$ is the model of the receptor of GABA_B_ current. In the simulation, the values of the external current of the TC neuron and maximal conductance from the RT neuron to the TC neuron ($I_{{GABA_{A} }}^{RT}$) are different values for modeling the normal state ($I_{ext}^{TC} = 5 \, \upmu {\text{A}} \; {\text{cm}}^{ - 2} ,\;\;\overline{g}_{{GABA_{A} }} = 0.65$ mS cm^−2^) and CAE state with an oscillatory spiking pattern ($I_{ext}^{TC} = 6 \, \upmu {\text{A}} \; {\text{cm}}^{ - 2} ,\;\;\overline{g}_{{GABA_{A} }} = 0.32$ mS cm^−2^)^25^.

**Table 1:** Synaptic current equations and their parameters for the neuron model.

Current	Equation	Dynamics and their equations	Parameters
**TC synapses**			
$I_{AMPA}^{CT}$	$\overline{g}_{AMPA} s_{AMPA} \left( {V_{TC} - E_{AMPA} } \right)$	$\frac{d}{dt}s_{AMPA} = \alpha_{AMPA} T_{AMPA} \left( {1 - s_{AMPA} } \right) - \beta_{AMPA} s_{AMPA}$ $T_{AMPA} = {{2.84} \mathord{\left/ {\vphantom {{2.84} {\left( {1 + e^{{{{\left( {2 - V_{CT} \left( {t - \tau_{1} } \right)} \right)} \mathord{\left/ {\vphantom {{\left( {2 - V_{CT} \left( {t - \tau_{1} } \right)} \right)} 5}} \right. \kern-0pt} 5}}} } \right)}}} \right. \kern-0pt} {\left( {1 + e^{{{{\left( {2 - V_{CT} \left( {t - \tau_{1} } \right)} \right)} \mathord{\left/ {\vphantom {{\left( {2 - V_{CT} \left( {t - \tau_{1} } \right)} \right)} 5}} \right. \kern-0pt} 5}}} } \right)}}$	$$\begin{gathered} \overline{g}_{AMPA} = 0.1 \hfill \\ E_{AMPA} = 0 \hfill \\ \alpha_{AMPA} = 0.94 \hfill \\ \beta_{AMPA} = 0.18 \hfill \\ \end{gathered}$$
$$I_{{GABA_{A} }}^{RT}$$	$$\overline{g}_{{GABA_{A} }} s_{{GABA_{A} }} \left( {V_{TC} - E_{{GABA_{A} }} } \right)$$	$$\frac{d}{dt}s_{{GABA_{A} }} = \alpha_{{GABA_{A} }} T_{{GABA_{A} }} \left( {1 - s_{{GABA_{A} }} } \right) - \beta_{{GABA_{A} }} s_{{GABA_{A} }}$$$$T_{{GABA_{A} }} = {{2.84} \mathord{\left/ {\vphantom {{2.84} {\left( {1 + e^{{{{\left( {2 - V_{RT} } \right)} \mathord{\left/ {\vphantom {{\left( {2 - V_{RT} } \right)} 5}} \right. \kern-0pt} 5}}} } \right)}}} \right. \kern-0pt} {\left( {1 + e^{{{{\left( {2 - V_{RT} } \right)} \mathord{\left/ {\vphantom {{\left( {2 - V_{RT} } \right)} 5}} \right. \kern-0pt} 5}}} } \right)}}$$	$$\begin{gathered} \overline{g}_{{GABA_{A} }} = 0.1 - 0.7 \hfill \\ E_{{GABA_{A} }} = - 85 \hfill \\ \alpha_{{GABA_{A} }} = 5 \hfill \\ \beta_{{GABA_{A} }} = 0.18 \hfill \\ \end{gathered}$$
$I_{{GABA_{B} }}^{RT}$	$$\begin{gathered} {{\overline{g}_{{GABA_{B} }} s_{{GABA_{B} }}^{4} } \mathord{\left/ {\vphantom {{\overline{g}_{{GABA_{B} }} s_{{GABA_{B} }}^{4} } {\left( {s_{{GABA_{B} }}^{4} + K_{d} } \right)}}} \right. \kern-0pt} {\left( {s_{{GABA_{B} }}^{4} + K_{d} } \right)}} \times \hfill \\ \left( {V_{TC} - E_{{GABA_{B} }} } \right) \hfill \\ \end{gathered}$$	$\frac{d}{dt}s_{{GABA_{B} }} = 0.18r_{{GABA_{B} }} - 0.034s_{{GABA_{B} }}$ $\frac{d}{dt}r_{{GABA_{B} }} = 0.5T_{{GABA_{B} }} \left( {1 - r_{{GABA_{B} }} } \right) - 0.0012r_{{GABA_{B} }}$ $T_{{GABA_{B} }} = {{2.84} \mathord{\left/ {\vphantom {{2.84} {\left( {1 + e^{{{{\left( {2 - V_{RT} } \right)} \mathord{\left/ {\vphantom {{\left( {2 - V_{RT} } \right)} 5}} \right. \kern-0pt} 5}}} } \right)}}} \right. \kern-0pt} {\left( {1 + e^{{{{\left( {2 - V_{RT} } \right)} \mathord{\left/ {\vphantom {{\left( {2 - V_{RT} } \right)} 5}} \right. \kern-0pt} 5}}} } \right)}}$	$$\begin{gathered} \overline{g}_{{GABA_{B} }} = 0.13793 \hfill \\ E_{{GABA_{B} }} = - 95 \hfill \\ K_{d} = 100 \hfill \\ \end{gathered}$$
**CT synapses**			
$I_{AMPA}^{TC}$	$\overline{g}_{AMPA} s_{AMPA} \left( {V_{CT} - E_{AMPA} } \right)$	$\frac{d}{dt}s_{AMPA} = \alpha_{AMPA} T_{AMPA} \left( {1 - s_{AMPA} } \right) - \beta_{AMPA} s_{AMPA}$ $T_{AMPA} = {{2.84} \mathord{\left/ {\vphantom {{2.84} {\left( {1 + e^{{{{\left( {2 - V_{TC} \left( {t - \tau_{2} } \right)} \right)} \mathord{\left/ {\vphantom {{\left( {2 - V_{TC} \left( {t - \tau_{2} } \right)} \right)} 5}} \right. \kern-0pt} 5}}} } \right)}}} \right. \kern-0pt} {\left( {1 + e^{{{{\left( {2 - V_{TC} \left( {t - \tau_{2} } \right)} \right)} \mathord{\left/ {\vphantom {{\left( {2 - V_{TC} \left( {t - \tau_{2} } \right)} \right)} 5}} \right. \kern-0pt} 5}}} } \right)}}$	$$\begin{gathered} \overline{g}_{AMPA} = 4.138 \hfill \\ E_{AMPA} = 0 \hfill \\ \alpha_{AMPA} = 0.94 \hfill \\ \beta_{AMPA} = 0.18 \hfill \\ \end{gathered}$$
**RT synapses**			
$$I_{{GABA_{A} }}^{RT}$$	$$\overline{g}_{{GABA_{A} }} s_{{GABA_{A} }} \left( {V_{RT} - E_{{GABA_{A} }} } \right)$$	$$\frac{d}{dt}s_{{GABA_{A} }} = \alpha_{{GABA_{A} }} T_{{GABA_{A} }} \left( {1 - s_{{GABA_{A} }} } \right) - \beta_{{GABA_{A} }} s_{{GABA_{A} }}$$$$T_{{GABA_{A} }} = {{2.84} \mathord{\left/ {\vphantom {{2.84} {\left( {1 + e^{{{{\left( {2 - V_{RT} } \right)} \mathord{\left/ {\vphantom {{\left( {2 - V_{RT} } \right)} 5}} \right. \kern-0pt} 5}}} } \right)}}} \right. \kern-0pt} {\left( {1 + e^{{{{\left( {2 - V_{RT} } \right)} \mathord{\left/ {\vphantom {{\left( {2 - V_{RT} } \right)} 5}} \right. \kern-0pt} 5}}} } \right)}}$$	$$\begin{gathered} \overline{g}_{{GABA_{A} }} = 6 \hfill \\ E_{{GABA_{A} }} = - 85 \hfill \\ \alpha_{{GABA_{A} }} = 5 \hfill \\ \beta_{{GABA_{A} }} = 0.18 \hfill \\ \end{gathered}$$
$I_{AMPA}^{TC}$	$\overline{g}_{AMPA} s_{AMPA} \left( {V_{RT} - E_{AMPA} } \right)$	$\frac{d}{dt}s_{AMPA} = \alpha_{AMPA} T_{AMPA} \left( {1 - s_{AMPA} } \right) - \beta_{AMPA} s_{AMPA}$ $T_{AMPA} = {{2.84} \mathord{\left/ {\vphantom {{2.84} {\left( {1 + e^{{{{\left( {2 - V_{TC} } \right)} \mathord{\left/ {\vphantom {{\left( {2 - V_{TC} } \right)} 5}} \right. \kern-0pt} 5}}} } \right)}}} \right. \kern-0pt} {\left( {1 + e^{{{{\left( {2 - V_{TC} } \right)} \mathord{\left/ {\vphantom {{\left( {2 - V_{TC} } \right)} 5}} \right. \kern-0pt} 5}}} } \right)}}$	$$\begin{gathered} \overline{g}_{AMPA} = 1.428 \hfill \\ E_{AMPA} = 0 \hfill \\ \alpha_{AMPA} = 0.94 \hfill \\ \beta_{AMPA} = 0.18 \hfill \\ \end{gathered}$$

### Control problem

The main objective of the controller is to force the membrane voltage of the TC, CT, and RT neurons in CAE condition to track the normal state of the model. To this end, the conductance-based model described in (1) is used as a CAE model (virtual patient). To implement AFTSMC by delivering the continuous DBS stimulation (control input) to suppress the oscillatory pattern of the neurons, the dynamics of the membrane voltage of the three-neuron CAE motif in (1) should be represented as a canonical MIMO nonlinear model with the following nonlinear differential equation:

3 $${\dot{\mathbf{x}}}(t) = {\mathbf{f}}({\mathbf{x}},t) + {\mathbf{f}}_{\tau } ({\mathbf{x}}_{\tau } ,t) + {\mathbf{G}}({\mathbf{x}},t) \cdot {\mathbf{u}}(t) + {\mathbf{d}}(t),$$

where ${\mathbf{x}} = [x_{1} , \ldots , \, x_{m} ]^{T} ,{\mathbf{x}}_{\tau } = [x_{1} (t - \tau ), \ldots , \, x_{m} (t - \tau )]^{T} ,m = 1,...,3$ is a measurable membrane potential of TC, CT, and RT neurons and ${\mathbf{u}} = [u_{1} , \ldots , u_{m} ]^{T}$ is the control input (continuous DBS pulses). ${\mathbf{d}}(t)$ denotes unknown external disturbances and uncertainties of the system, which is bounded with a positive unknown value, i.e., $\left\| {{\mathbf{d}}(t)} \right\| < \upsilon$. The unknown continuous vector functions ${\mathbf{f}}({\mathbf{x}},t),{\mathbf{f}}_{\tau } ({\mathbf{x}}_{\tau } ,t)$ and ${\mathbf{G}}({\mathbf{x}},t)$ are defined as

4 $$\begin{aligned} {\mathbf{f}}({\mathbf{x}},t) & = \left[ {f_{{1}} {(}{\mathbf{x}},t{), } \ldots {, }f_{{\text{m}}} {(}{\mathbf{x}},t{)}} \right]^{T} \hfill \\ {\mathbf{f}}_{\tau } ({\mathbf{x}}_{\tau } ,t) & = \left[ {f_{{\tau_{{1}} }} {(}{\mathbf{x}}_{\tau } ,t{), } \ldots {, }f_{{\tau_{{\text{m}}} }} {(}{\mathbf{x}}_{\tau } ,t{)}} \right]^{T} \hfill \\ \end{aligned}$$

5 $${\mathbf{G}}({\mathbf{x}},t) = \left[ {\begin{array}{*{20}c} {g_{11} ({\mathbf{x}},t)} & \cdots & {g_{1m} ({\mathbf{x}},t)} \\ \vdots & \ddots & \vdots \\ {g_{m1} ({\mathbf{x}},t)} & \cdots & {g_{mm} ({\mathbf{x}},t)} \\ \end{array} } \right].$$

#### **Assumption 1**

${\mathbf{G}}({\mathbf{x}},t)$ is a positive definite matrix that is conditioned to ${\mathbf{G}}({\mathbf{x}},t) > \chi_{0} {\mathbf{I}}_{m}$, where $\chi_{0}$ is a positive real parameter and ${\mathbf{I}}_{m}$ is an $m \times m$ identity matrix.

#### **Assumption 2**

The desired trajectory ${\mathbf{x}}_{d} (t)$ is a continuous membrane potential of TC, CT, and RT neurons in a normal condition that is measurable and its first-order dynamic exists.

#### **Assumption 3**

The time delay $\tau$ is known and measurable.

The tracking error of the conductance-based model is defined as $e_{i} = x_{{d_{i} }} - x_{i}$. To design AFTSMC the nonsingular terminal sliding surface is considered with the following function:

6 $$s_{i} (t) = \int\limits_{0}^{t} {e_{i} (t)dt} + \sigma \left| {e_{i} (t)} \right|^{\eta } sign(e_{i} (t)), \quad i = 1,...,m$$

where $\sigma > 0$ and $1 < \eta < 2$ are positive design numbers. If the initial states of the system are outside the sliding surface, a fast TSM-type reaching law is designed to ensure the convergence of the system states to the sliding surface in finite time as follows:

7 $${\dot{\mathbf{s}}} = - {\mathbf{K}}_{{\mathbf{1}}} {\mathbf{s}} - {\mathbf{K}}_{{\mathbf{2}}} \left| {\mathbf{s}} \right|^{\rho } sign({\mathbf{s}}),$$

where the matrices ${\mathbf{K}}_{1} = diag(k_{11} ,...,k_{1m} ) > {\mathbf{0}}_{m \times m}$ and ${\mathbf{K}}_{2} = diag(k_{21} ,...,k_{2m} ) > {\mathbf{0}}_{m \times m}$ are design control parameters and $0 < \rho < 1$. According to Eq. (6), the first derivative of the sliding manifold is

8 $${\dot{\mathbf{s}}} = {\mathbf{e}} + \eta \sigma diag(\left| {\mathbf{e}} \right|^{\eta - 1} ){\dot{\mathbf{e}}}.$$

The control input is designed in the following form:

9 $${\mathbf{u}}(t) = {\mathbf{G}}^{ - 1} ({\mathbf{x}},t)( - {\mathbf{f}}({\mathbf{x}},t) - {\mathbf{f}}_{\tau } ({\mathbf{x}}_{\tau } ,t) - {\mathbf{d}}(t) + {\dot{\mathbf{x}}}_{d} (t) + \frac{1}{\eta \sigma }\left| {\mathbf{e}} \right|^{2 - \eta } sign({\mathbf{e}}) + {\mathbf{K}}_{{\mathbf{1}}} {\mathbf{s}} + {\mathbf{K}}_{{\mathbf{2}}} \left| {\mathbf{s}} \right|^{\rho } sign({\mathbf{s}})).$$

#### **Lemma 1**

*If a Lyapunov description of finite-time stability is given as follows:*

10 $$\dot{V}({\mathbf{x}}) + \alpha_{1} V({\mathbf{x}}) + \alpha_{2} V^{\lambda } ({\mathbf{x}}) \le 0,$$

*where* $\alpha_{1} ,\alpha_{2} > 0$ *and* $0 < \lambda < 1$, *then, the settling time is calculated by the following equation*^61^:

11 $$T \le \frac{1}{{\alpha_{1} \left( {1 - \lambda } \right)}}\ln \frac{{\alpha_{1} V({\mathbf{x}}_{0} )^{1 - \lambda } + \alpha_{2} }}{{\alpha_{2} }}.$$

#### **Lemma 2**

*If* $a_{1} ,a_{2} ,...,a_{n}$ *are all positive numbers, and* $0 < p \le 2$, *then the following inequality always* *maintains*^61^:

12 $$\left( {a_{1}^{2} + \cdots + a_{n}^{2} } \right)^{p} \le \left( {a_{1}^{p} + \cdots + a_{n}^{p} } \right)^{2} .$$

#### **Theorem 1**

*Consider the dynamical MIMO system with delay in* (3), *the assumptions* (2) *and* (3) *are held and the nonlinear dynamics of the system with the external disturbances are assumed to be known. The integral-based terminal switching surface is considered as* (6), *the control input is designed by* (9), *and the finite-time reachability condition to the sliding surface is ensured with* (7). *If the dynamic states of the model with any initial conditions are outside the sliding surface, then the finite-time convergence of the model dynamics to the switching surface* $s_{i} = 0$ *is guaranteed. On the switching surface, the tracking error decreases to zero in a finite time.*

#### *Proof*

Consider the first dynamic of the tracking error with ${\dot{\mathbf{e}}}(t) = {\dot{\mathbf{x}}}_{{\mathbf{d}}} (t) - {\dot{\mathbf{x}}}(t)$, the first derivative of the sliding surface vector using (8) is rewritten as

13 $$ \begin{aligned} {\dot{\mathbf{s}}} & = {\mathbf{e}} + \eta \sigma diag(\left| {\mathbf{e}} \right|^{\eta - 1} )\left( {{\dot{\mathbf{x}}}_{{\mathbf{d}}} (t) - {\dot{\mathbf{x}}} (t)} \right) \hfill \\ {\dot{\mathbf{s}}} & = {\mathbf{e}} + \eta \sigma diag(\left| {\mathbf{e}} \right|^{\eta - 1} )\left( {{\dot{\mathbf{x}}}_{{\mathbf{d}}} (t) - {\mathbf{f}}({\mathbf{x}},t) - {\mathbf{f}}_{\tau } ({\mathbf{x}}_{\tau } ,t) - {\mathbf{G}}({\mathbf{x}},t) \cdot {\mathbf{u}}(t) - {\mathbf{d}}(t)} \right) \hfill \\ {\dot{\mathbf{s}}} & = {\mathbf{e}} + \eta \sigma diag(\left| {\mathbf{e}} \right|^{\eta - 1} )\left( {{\dot{\mathbf{x}}}_{{\mathbf{d}}} (t) - {\mathbf{f}}({\mathbf{x}},t) - {\mathbf{f}}_{\tau } ({\mathbf{x}}_{\tau } ,t) - {\mathbf{d}}(t)} \right) - \eta \sigma diag(\left| {\mathbf{e}} \right|^{\eta - 1} ){\mathbf{G}}({\mathbf{x}},t) \hfill \\ & \quad \times {\mathbf{G}}^{ - 1} ({\mathbf{x}},t)( - {\mathbf{f}}({\mathbf{x}},t) - {\mathbf{f}}_{\tau } ({\mathbf{x}}_{\tau } ,t) - {\mathbf{d}}(t) + {\dot{\mathbf{x}}}_{d} (t) + \frac{1}{\eta \sigma }\left| {\mathbf{e}} \right|^{2 - \eta } sign({\mathbf{e}}) \\ & \quad + {\mathbf{K}}_{{\mathbf{1}}} {\mathbf{s}} + {\mathbf{K}}_{{\mathbf{2}}} \left| {\mathbf{s}} \right|^{\rho } sign({\mathbf{s}})) \hfill \\ {\dot{\mathbf{s}}} & = - \eta \sigma diag(\left| {\mathbf{e}} \right|^{\eta - 1} )\left( {{\mathbf{K}}_{{\mathbf{1}}} {\mathbf{s}} + {\mathbf{K}}_{{\mathbf{2}}} \left| {\mathbf{s}} \right|^{\rho } sign({\mathbf{s}})} \right). \hfill \\ \end{aligned}$$

By considering the Lyapunov function $V = 0.5{\mathbf{s}}^{T} {\mathbf{s}}$ and substituting (13) in its first dynamic, it yields

14 $$\dot{V} = - {\mathbf{s}}^{T} {\overline{\mathbf{K}}}_{1} {\mathbf{s}} - {\mathbf{s}}^{T} {\overline{\mathbf{K}}}_{2} \left| {\mathbf{s}} \right|^{\rho } sign({\mathbf{s}}),$$

where ${\overline{\mathbf{K}}}_{1} = \eta \sigma diag(\left| {\mathbf{e}} \right|^{\eta - 1} ){\mathbf{K}}_{1}$ and ${\overline{\mathbf{K}}}_{2} = \eta \sigma diag(\left| {\mathbf{e}} \right|^{\eta - 1} ){\mathbf{K}}_{2}$ are modified gain matrices. Using Lemma 2, we have

15 $$\dot{V} \le - 2\overline{k}_{1} V - 2^{{\frac{1 + \rho }{2}}} \overline{k}_{2} V^{{\frac{1 + \rho }{2}}} ,$$

where $\overline{k}_{1} = min_{j} \left\{ {\overline{k}_{1j} } \right\} > 0$ and $\overline{k}_{2} = min_{j} \left\{ {\overline{k}_{2j} } \right\} > 0,j = 1,...,m$ are minimum eigenvalues of matrices ${\overline{\mathbf{K}}}_{1}$ and ${\overline{\mathbf{K}}}_{2}$, respectively, and $0.5 < {{(1 + \rho )} \mathord{\left/ {\vphantom {{(1 + \rho )} 2}} \right. \kern-0pt} 2} < 1$ to satisfy the condition of Lemma 2. The finite-time convergence to the sliding surface ${\mathbf{s}} = 0$ is guaranteed based on the criterion presented in Lemma 2 and using Eq. (11), the finite time for reaching the sliding surface is as follows:

16 $$t_{r} \le \frac{1}{{\overline{k}_{1} \left( {1 - \rho } \right)}}\ln \frac{{\overline{k}_{1} V^{{\frac{1 - \rho }{2}}} ({\mathbf{x}}_{0} ) + 2^{{\frac{\rho - 1}{2}}} \overline{k}_{2} }}{{2^{{\frac{\rho - 1}{2}}} \overline{k}_{2} }}.$$

After the reaching of the states to the sliding surface ${\mathbf{s}} = 0$, according to the terminal sliding surface considered in (4), the convergence time required to achieve the global stable attractor of (6) ($e_{i} = 0$) for any initial condition $x_{i} (t_{{r_{i} }} )$ is finite and is computed as follows:

17 $$t_{{s_{i} }} = \frac{\sigma \eta }{{\eta - 1}}\left| {x_{i} (t_{{r_{i} }} )} \right|^{\eta - 1} .$$

This completes the proof of Theorem 1.

### Adaptive fuzzy terminal sliding mode control (AFTSMC)

Since in the real DBS systems, the dynamics of nonlinear functions ${\mathbf{f}}({\mathbf{x}},t) + {\mathbf{f}}_{\tau } ({\mathbf{x}}_{\tau } ,t) + {\mathbf{d}}(t)$ and ${\mathbf{G}}({\mathbf{x}},t)$ are unknown, the control input (9) cannot implement. In the current study, to eliminate the limitation, FLS is applied to approximate the unknown nonlinear dynamics of the system. If ${\hat{\mathbf{f}}}({\mathbf{x}},{{\varvec{\uppsi}}}_{f}^{t} ),{\hat{\mathbf{f}}}_{\tau } ({\mathbf{x}},{{\varvec{\uppsi}}}_{{f_{\tau } }}^{t} )$ and ${\hat{\mathbf{G}}}({\mathbf{x}},{{\varvec{\uppsi}}}_{g}^{t} )$ are the fuzzy estimations of ${\mathbf{f}}({\mathbf{x}},t),{\mathbf{f}}_{\tau } ({\mathbf{x}}_{\tau } ,t)$ and ${\mathbf{G}}({\mathbf{x}},t)$, respectively, the control input (9) can be modified as follows:

18 $$\begin{aligned} {\mathbf{u}}_{c} (t) & = \frac{{{\hat{\mathbf{G}}}^{T} ({\mathbf{x}},{{\varvec{\uppsi}}}_{g}^{t} )}}{{\varepsilon_{0} {\mathbf{I}}_{m} + {\hat{\mathbf{G}}}({\mathbf{x}},{{\varvec{\uppsi}}}_{g}^{t} ){\hat{\mathbf{G}}}^{T} ({\mathbf{x}},{{\varvec{\uppsi}}}_{g}^{t} )}} \hfill \\ & \quad \times \left( { - {\hat{\mathbf{f}}}({\mathbf{x}},{{\varvec{\uppsi}}}_{f}^{t} ) - {\hat{\mathbf{f}}}_{\tau } ({\mathbf{x}}_{\tau } ,{{\varvec{\uppsi}}}_{{f_{\tau } }}^{t} ) + {\dot{\mathbf{x}}}_{d} (t) + \frac{1}{\eta \sigma }\left| {\mathbf{e}} \right|^{2 - \eta } sign({\mathbf{e}}) + {\mathbf{K}}_{{\mathbf{1}}} {\mathbf{s}} + {\mathbf{K}}_{{\mathbf{2}}} \left| {\mathbf{s}} \right|^{\rho } sign({\mathbf{s}})} \right), \hfill \\ \end{aligned}$$

where $\varepsilon_{0} > 0$ is a very small parameter. In (18), the regular form ${\hat{\mathbf{G}}}({\mathbf{x}},{{\varvec{\uppsi}}}_{g}^{t} )^{ - 1}$ is used to overcome the singularity of ${\hat{\mathbf{G}}}({\mathbf{x}},{{\varvec{\uppsi}}}_{g}^{t} )$ and thus the well definition of control input (18). A detailed description of FLS is provided in Appendix B of the Supplementary Materials. Let us define the minimum approximation errors of the fuzzy estimator as

19 $$\begin{aligned} {{\varvec{\upvarepsilon}}}_{f}^{{}} ({\mathbf{x}},t) & = {\mathbf{f}}({\mathbf{x}},t) - {\hat{\mathbf{f}}}^{*} ({\mathbf{x}},{{\varvec{\uppsi}}}_{f}^{*} ) = \left[ {\begin{array}{*{20}c} {\varepsilon_{{f_{1} }} } & \cdots & {\varepsilon_{{f_{m} }} } \\ \end{array} } \right]^{T} \hfill \\ {{\varvec{\upvarepsilon}}}_{{f_{\tau } }}^{{}} ({\mathbf{x}}_{\tau } ,t) & = {\mathbf{f}}_{\tau } ({\mathbf{x}}_{\tau } ,t) - {\hat{\mathbf{f}}}_{\tau }^{*} ({\mathbf{x}}_{\tau } ,{{\varvec{\uppsi}}}_{{f_{\tau } }}^{*} ) = \left[ {\begin{array}{*{20}c} {\varepsilon_{{f_{{\tau_{1} }} }} } & \cdots & {\varepsilon_{{f_{{\tau_{m} }} }} } \\ \end{array} } \right]^{T} \hfill \\ \end{aligned}$$

20 $${{\varvec{\upvarepsilon}}}_{g}^{{}} ({\mathbf{x}},t) = {\mathbf{G}}({\mathbf{x}},t) - {\mathbf{G}}^{*} ({\mathbf{x}},{{\varvec{\uppsi}}}_{g}^{*} ) = \left[ {\begin{array}{*{20}c} {\varepsilon_{{g_{11} }} } & \cdots & {\varepsilon_{{g_{1m} }} } \\ \vdots & \ddots & \vdots \\ {\varepsilon_{{g_{m1} }} } & \cdots & {\varepsilon_{{g_{mm} }} } \\ \end{array} } \right],$$

where ${\hat{\mathbf{f}}}^{*} ({\mathbf{x}},{{\varvec{\uppsi}}}_{f}^{*} )$, ${\hat{\mathbf{f}}}_{\tau }^{*} ({\mathbf{x}}_{\tau } ,{{\varvec{\uppsi}}}_{{f_{\tau } }}^{*} )$, and ${\hat{\mathbf{G}}}^{*} ({\mathbf{x}},{{\varvec{\uppsi}}}_{g}^{*} )$ are the optimal estimated functions. We have used the following inequalities to limit the values of the minimum approximation errors:

21 $$\left| {\varepsilon_{{f_{i} }}^{{}} ({\mathbf{x}},t)} \right| \le \overline{\varepsilon }_{{f_{i} }}^{{}} ,\left| {\varepsilon_{{f_{{\tau_{i} }} }}^{{}} ({\mathbf{x}}_{\tau } ,t)} \right| \le \overline{\varepsilon }_{{f_{{\tau_{i} }} }}^{{}} ,\left| {\varepsilon_{{g_{ij} }}^{{}} ({\mathbf{x}},t)} \right| \le \overline{\varepsilon }_{g}^{{}} , \quad \forall {\mathbf{x}} \in {\text{D}}_{{\text{x}}}$$

where $\overline{\varepsilon }_{{f_{i} }}^{{}} ,\overline{\varepsilon }_{{f_{{\tau_{i} }} }}^{{}}$ and $\overline{\varepsilon }_{g}^{{}}$ are unknown small values and will be estimated with adaptive rules. To online designation of ${\hat{\mathbf{f}}}({\mathbf{x}},{{\varvec{\uppsi}}}_{f}^{t} ),{\hat{\mathbf{f}}}_{\tau } ({\mathbf{x}}_{\tau } ,{{\varvec{\uppsi}}}_{{f_{\tau } }}^{t} )$, and ${\hat{\mathbf{G}}}({\mathbf{x}},{{\varvec{\uppsi}}}_{g}^{t} )$, the adaptive parameters of the fuzzy estimator in (S.24) and (S.25) should be updated online with the following adaptation rules:

22 $$\begin{aligned} \dot{\psi }_{{f_{i} }}^{t} & = - \kappa_{{f_{i} }} \sigma \eta \left| {e_{i} } \right|^{\eta - 1} \zeta_{{f_{i} }} ({\mathbf{x}})s_{i} \hfill \\ \dot{\psi }_{{f_{{\tau_{i} }} }}^{t} & = - \kappa_{{f_{{\tau_{i} }} }} \sigma \eta \left| {e_{i} } \right|^{\eta - 1} \zeta_{{f_{{\tau_{i} }} }} ({\mathbf{x}}_{\tau } )s_{i} \hfill \\ \end{aligned}$$

23 $$\dot{\psi }_{{g_{ij} }}^{t} = - \kappa_{{g_{ij} }} \sigma \eta \left| {e_{i} } \right|^{\eta - 1} \zeta_{{g_{ij} }} ({\mathbf{x}})s_{i} u_{{c_{j} }} ,$$

where $\kappa_{{f_{i} }} ,\kappa_{{f_{{\tau_{i} }} }} > 0$ and $\kappa_{{g_{ij} }} > 0$. $\zeta_{{f_{{_{i} }} }} {\mathbf{(x)}}$, $\zeta_{{f_{{_{{\tau_{i} }} }} }} {\mathbf{(x}}_{\tau } {\mathbf{)}}$, and $\zeta_{{g_{ij} }} {\mathbf{(x)}}$ denote fuzzy basis vector fixed with the designer (see equation S.23 in Appendix B of the Supplementary Materials). To guarantee the stability of the closed-loop system in the presence of approximation errors, external disturbances, and regularized inverse ${\hat{\mathbf{G}}}({\mathbf{x}},{{\varvec{\uppsi}}}_{g}^{t} )$, the corrected control signal ${\mathbf{u}}_{r} (t)$ is added to the control input (18) as a robustifying term

24 $${\mathbf{u}}(t) = {\mathbf{u}}_{c} (t) + {\mathbf{u}}_{r} (t),$$

where ${\mathbf{u}}_{r} (t)$ is designed as follows﻿:

25 $$ {\mathbf{u}}_{r} (t) = \frac{{{\mathbf{s}}\left| {{\mathbf{s}}^{T} } \right|({\hat{\mathbf{\varepsilon }}}_{f} + {\hat{\mathbf{\varepsilon }}}_{{f_{\tau } }} + \hat{\varepsilon }_{g} \left| {{\mathbf{u}}_{c} } \right| + {\hat{\mathbf{d}}} + \left| {{\mathbf{u}}_{0} } \right|)}}{{\sigma_{0} \left\| {\mathbf{s}} \right\|^{2} + \Upsilon }}, $$

where ${\hat{\mathbf{\varepsilon }}}_{f}$, ${\hat{\mathbf{\varepsilon }}}_{{f_{\tau } }}$, and $\hat{\varepsilon }_{g}$ are the estimation of the upper bound of the minimum approximation errors ${\overline{\mathbf{\varepsilon }}}_{f}^{{}}$, ${\overline{\mathbf{\varepsilon }}}_{{f_{\tau } }}^{{}}$, and $\overline{\varepsilon }_{g}$, respectively, ${\hat{\mathbf{d}}}$ is the estimation of the bound of the external disturbance term, and $\sigma_{0} > 0$. $\Upsilon$ is an adaptive small parameter designed with

26 $$\begin{aligned} \dot{\Upsilon } & = - \kappa_{0} \frac{{\left| {{\mathbf{s}}^{T} } \right|\eta \sigma diag(\left| {\mathbf{e}} \right|^{\eta - 1} )({\hat{\mathbf{\varepsilon }}}_{f} + {\hat{\mathbf{\varepsilon }}}_{{f_{\tau } }} + \hat{\varepsilon }_{g} \left| {{\mathbf{u}}_{c} } \right| + {\hat{\mathbf{d}}} + \left| {{\mathbf{u}}_{0} } \right|)}}{{\sigma_{0} \left\| {\mathbf{s}} \right\|^{2} + \Upsilon }} \hfill \\ {\dot{\hat{\mathbf{\varepsilon }}}}_{f} & = \gamma_{0} \eta \sigma diag(\left| {\mathbf{e}} \right|^{\eta - 1} )\left| {\mathbf{s}} \right| \hfill \\ {\dot{\hat{\mathbf{\varepsilon }}}}_{{f_{\tau } }} & = \gamma_{1} \eta \sigma diag(\left| {\mathbf{e}} \right|^{\eta - 1} )\left| {\mathbf{s}} \right| \hfill \\ \dot{\hat{\varepsilon }}_{g} & = \gamma_{2} \eta \sigma \left| {{\mathbf{s}}^{T} } \right|diag(\left| {\mathbf{e}} \right|^{\eta - 1} )\left| {{\mathbf{u}}_{c} } \right| \hfill \\ {\dot{\hat{\mathbf{d}}}} & = \gamma_{3} \eta \sigma diag(\left| {\mathbf{e}} \right|^{\eta - 1} )\left| {\mathbf{s}} \right|, \hfill \\ \end{aligned}$$

where $\kappa_{0} ,\gamma_{0} ,\gamma_{1} ,\gamma_{2} ,\gamma_{3}$ are all positive parameters. In (25) ${\mathbf{u}}_{0} (t)$ is designed as follows:

27 $$\begin{aligned} {\mathbf{u}}_{0} (t) & = \varepsilon_{0} (\varepsilon_{0} {\mathbf{I}}_{m} + {\hat{\mathbf{G}}}({\mathbf{x}},{{\varvec{\uppsi}}}_{g}^{t} ){\hat{\mathbf{G}}}^{T} ({\mathbf{x}},{{\varvec{\uppsi}}}_{g}^{t} ))^{ - 1} \hfill \\ & \quad \times \left( { - {\hat{\mathbf{f}}}({\mathbf{x}},{{\varvec{\uppsi}}}_{f}^{t} ) - {\hat{\mathbf{f}}}_{\tau } ({\mathbf{x}}_{\tau } ,{{\varvec{\uppsi}}}_{{f_{\tau } }}^{t} ) + {\dot{\mathbf{x}}}_{d} (t) + \frac{1}{\eta \sigma }\left| {\mathbf{e}} \right|^{2 - \eta } sign({\mathbf{e}}) + {\mathbf{K}}_{{\mathbf{1}}} {\mathbf{s}} + {\mathbf{K}}_{{\mathbf{2}}} \left| {\mathbf{s}} \right|^{\rho } sign({\mathbf{s}})} \right). \hfill \\ \end{aligned}$$

#### **Theorem 2**

*Consider the dynamical MIMO nonlinear model with delay in* (3), *the assumptions* (1)–(3) *are hold, and the nonlinear time-varying dynamics*${\mathbf{f}}({\mathbf{x}},t),{\mathbf{f}}_{\tau } ({\mathbf{x}}_{\tau } ,t)$ *and* ${\mathbf{G(x}},t{\mathbf{)}}$ *are approximated using FLS with* (S.24) *and* (S.25). *If the control input is designed as* (24) *with the adaptation laws in* (22), (23), *and* (26), *then the following results are guaranteed:*

1. *All signals in the closed-loop system are bounded*
2. *the sliding variable decreases to the neighborhood of zero as follows:*
  28 $$\left\| {\mathbf{s}} \right\| \le {{\left( \begin{gathered} \left\| {{\mathbf{f}}^{*} ({\mathbf{x}},{{\varvec{\uppsi}}}_{f}^{*} ) - {\hat{\mathbf{f}}}({\mathbf{x}},{{\varvec{\uppsi}}}_{f}^{t} )} \right\| + \left\| {{\mathbf{f}}_{\tau }^{*} ({\mathbf{x}}_{\tau } ,{{\varvec{\uppsi}}}_{{f_{\tau } }}^{*} ) - {\hat{\mathbf{f}}}_{\tau } ({\mathbf{x}}_{\tau } ,{{\varvec{\uppsi}}}_{{f_{\tau } }}^{t} )} \right\| + \left\| {{\mathbf{G}}^{*} ({\mathbf{x}},{{\varvec{\uppsi}}}_{g}^{*} ) - {\hat{\mathbf{G}}}({\mathbf{x}},{{\varvec{\uppsi}}}_{{f_{2} }}^{t} )} \right\|\left\| {{\mathbf{u}}_{c} } \right\| + \hfill \\ \left\| {sign({\mathbf{s}}^{T} )} \right\|\left\| {{\tilde{\mathbf{\varepsilon }}}_{f} ({\mathbf{x}}) + {\tilde{\mathbf{\varepsilon }}}_{{f_{\tau } }} ({\mathbf{x}}) + {\tilde{\mathbf{d}}}({\mathbf{x}}) + \tilde{\varepsilon }_{g} ({\mathbf{x}})\left| {{\mathbf{u}}_{c} } \right|} \right\| + \left\| {\Upsilon sign({\mathbf{s}}^{T} )} \right\|\left\| {\frac{{({\hat{\mathbf{\varepsilon }}}_{f} + {\hat{\mathbf{\varepsilon }}}_{{f_{\tau } }} + \hat{\varepsilon }_{g} \left| {{\mathbf{u}}_{c} } \right| + {\hat{\mathbf{d}}} + \left| {{\mathbf{u}}_{0} } \right|)}}{{\sigma_{0} \left\| {\mathbf{s}} \right\|^{2} + \Upsilon }}} \right\| \hfill \\ \end{gathered} \right)} \mathord{\left/ {\vphantom {{\left( \begin{gathered} \left\| {{\mathbf{f}}^{*} ({\mathbf{x}},{{\varvec{\uppsi}}}_{f}^{*} ) - {\hat{\mathbf{f}}}({\mathbf{x}},{{\varvec{\uppsi}}}_{f}^{t} )} \right\| + \left\| {{\mathbf{f}}_{\tau }^{*} ({\mathbf{x}}_{\tau } ,{{\varvec{\uppsi}}}_{{f_{\tau } }}^{*} ) - {\hat{\mathbf{f}}}_{\tau } ({\mathbf{x}}_{\tau } ,{{\varvec{\uppsi}}}_{{f_{\tau } }}^{t} )} \right\| + \left\| {{\mathbf{G}}^{*} ({\mathbf{x}},{{\varvec{\uppsi}}}_{g}^{*} ) - {\hat{\mathbf{G}}}({\mathbf{x}},{{\varvec{\uppsi}}}_{{f_{2} }}^{t} )} \right\|\left\| {{\mathbf{u}}_{c} } \right\| + \hfill \\ \left\| {sign({\mathbf{s}}^{T} )} \right\|\left\| {{\tilde{\mathbf{\varepsilon }}}_{f} ({\mathbf{x}}) + {\tilde{\mathbf{\varepsilon }}}_{{f_{\tau } }} ({\mathbf{x}}) + {\tilde{\mathbf{d}}}({\mathbf{x}}) + \tilde{\varepsilon }_{g} ({\mathbf{x}})\left| {{\mathbf{u}}_{c} } \right|} \right\| + \left\| {\Upsilon sign({\mathbf{s}}^{T} )} \right\|\left\| {\frac{{({\hat{\mathbf{\varepsilon }}}_{f} + {\hat{\mathbf{\varepsilon }}}_{{f_{\tau } }} + \hat{\varepsilon }_{g} \left| {{\mathbf{u}}_{c} } \right| + {\hat{\mathbf{d}}} + \left| {{\mathbf{u}}_{0} } \right|)}}{{\sigma_{0} \left\| {\mathbf{s}} \right\|^{2} + \Upsilon }}} \right\| \hfill \\ \end{gathered} \right)} {k_{1} }}} \right. \kern-0pt} {k_{1} }} = \delta_{1} .$$
  29 $$\begin{gathered} \left\| {\mathbf{s}} \right\| \le \left( {{{\left( \begin{gathered} \left\| {{\mathbf{f}}^{*} ({\mathbf{x}},{{\varvec{\uppsi}}}_{f}^{*} ) - {\hat{\mathbf{f}}}({\mathbf{x}},{{\varvec{\uppsi}}}_{f}^{t} )} \right\| + \left\| {{\mathbf{f}}_{\tau }^{*} ({\mathbf{x}}_{\tau } ,{{\varvec{\uppsi}}}_{{f_{\tau } }}^{*} ) - {\hat{\mathbf{f}}}_{\tau } ({\mathbf{x}}_{\tau } ,{{\varvec{\uppsi}}}_{{f_{\tau } }}^{t} )} \right\| + \left\| {{\mathbf{G}}^{*} ({\mathbf{x}},{{\varvec{\uppsi}}}_{g}^{*} ) - {\hat{\mathbf{G}}}({\mathbf{x}},{{\varvec{\uppsi}}}_{{f_{2} }}^{t} )} \right\|\left\| {{\mathbf{u}}_{c} } \right\| + \hfill \\ \left\| {sign({\mathbf{s}}^{T} )} \right\|\left\| {{\tilde{\mathbf{\varepsilon }}}_{f} ({\mathbf{x}}) + {\tilde{\mathbf{\varepsilon }}}_{{f_{\tau } }} ({\mathbf{x}}) + {\tilde{\mathbf{d}}}({\mathbf{x}}) + \tilde{\varepsilon }_{g} ({\mathbf{x}})\left| {{\mathbf{u}}_{c} } \right|} \right\| + \left\| {\Upsilon sign({\mathbf{s}}^{T} )} \right\|\left\| {\frac{{({\hat{\mathbf{\varepsilon }}}_{f} + {\hat{\mathbf{\varepsilon }}}_{{f_{\tau } }} + \hat{\varepsilon }_{g} \left| {{\mathbf{u}}_{c} } \right| + {\hat{\mathbf{d}}} + \left| {{\mathbf{u}}_{0} } \right|)}}{{\sigma_{0} \left\| {\mathbf{s}} \right\|^{2} + \Upsilon }}} \right\| \hfill \\ \end{gathered} \right)} \mathord{\left/ {\vphantom {{\left( \begin{gathered} \left\| {{\mathbf{f}}^{*} ({\mathbf{x}},{{\varvec{\uppsi}}}_{f}^{*} ) - {\hat{\mathbf{f}}}({\mathbf{x}},{{\varvec{\uppsi}}}_{f}^{t} )} \right\| + \left\| {{\mathbf{f}}_{\tau }^{*} ({\mathbf{x}}_{\tau } ,{{\varvec{\uppsi}}}_{{f_{\tau } }}^{*} ) - {\hat{\mathbf{f}}}_{\tau } ({\mathbf{x}}_{\tau } ,{{\varvec{\uppsi}}}_{{f_{\tau } }}^{t} )} \right\| + \left\| {{\mathbf{G}}^{*} ({\mathbf{x}},{{\varvec{\uppsi}}}_{g}^{*} ) - {\hat{\mathbf{G}}}({\mathbf{x}},{{\varvec{\uppsi}}}_{{f_{2} }}^{t} )} \right\|\left\| {{\mathbf{u}}_{c} } \right\| + \hfill \\ \left\| {sign({\mathbf{s}}^{T} )} \right\|\left\| {{\tilde{\mathbf{\varepsilon }}}_{f} ({\mathbf{x}}) + {\tilde{\mathbf{\varepsilon }}}_{{f_{\tau } }} ({\mathbf{x}}) + {\tilde{\mathbf{d}}}({\mathbf{x}}) + \tilde{\varepsilon }_{g} ({\mathbf{x}})\left| {{\mathbf{u}}_{c} } \right|} \right\| + \left\| {\Upsilon sign({\mathbf{s}}^{T} )} \right\|\left\| {\frac{{({\hat{\mathbf{\varepsilon }}}_{f} + {\hat{\mathbf{\varepsilon }}}_{{f_{\tau } }} + \hat{\varepsilon }_{g} \left| {{\mathbf{u}}_{c} } \right| + {\hat{\mathbf{d}}} + \left| {{\mathbf{u}}_{0} } \right|)}}{{\sigma_{0} \left\| {\mathbf{s}} \right\|^{2} + \Upsilon }}} \right\| \hfill \\ \end{gathered} \right)} {k_{2} }}} \right. \kern-0pt} {k_{2} }}} \right)^{1/\rho } \hfill \\ \, = \delta_{2} , \hfill \\ \end{gathered}$$
  *where* $k_{1}$ *and* $k_{2}$ *denote the minimum eigenvalues of matrices* ${\mathbf{K}}_{1}$ *and* ${\mathbf{K}}_{2}$*, respectively.* (28) *and* (29), *confirm that the region* $\left\| {\mathbf{s}} \right\| \le \delta = \min (\delta_{1} ,\delta_{2} )$ *will be achieved in finite time and then, the tracking error decreases finite time to a boundary layer*
  30 $$\left| {e_{i} (t)} \right| \le \left( {\frac{\delta }{\sigma }} \right)^{{\frac{1}{\eta }}} , \, i = 1,...,m.$$

*The proof is given in* Appendix C*of the Supplementary Materials*.

#### *Remark 1*

According to the bounds of (28) and (29), the matrices ${\mathbf{K}}_{1}$ and ${\mathbf{K}}_{2}$ should be large enough to decrease the boundary $\delta$. This increases the amplitude of the control input (pulse width or pulse amplitude of a DBS pulse) which limits the implementation of the controller in a real FES system.

#### *Remark 2*

The term $sig({\mathbf{s}})^{\rho }$ in the reachability law embedded in control input (18) and $\eta$ in sliding surface (6) are considered a bridge between linear SMC ($\rho \to 1,\eta \to 1$) and TSMC. These parameters should be selected appropriately to guarantee the finite-time convergence of the tracking error to the boundary layer and to achieve the control input without any singularity or chattering.

#### *Remark 3*

In this paper, to satisfy the sufficient controllability condition of (3) it was assumed that the gain matrix of the control input is positive definite. In clinical applications of the closed-loop DBS system, if the positive definite ${\mathbf{G}}({\mathbf{x}},t)$ do not exist, the proposed control scheme can be extended to the system by multiplying the regular matrix ${\mathbf{B}}({\mathbf{x}})$ by the matrix ${\mathbf{G}}({\mathbf{x}},t)$ such that ${\mathbf{G^{\prime}}}({\mathbf{x}},t) = {\mathbf{G}}({\mathbf{x}},t){\mathbf{B}}({\mathbf{x}})$ is positive definite and hence, the new control signal ${\mathbf{u^{\prime}}}({\mathbf{x}}) = {\mathbf{B}}({\mathbf{x}}){\mathbf{u}}({\mathbf{x}})$ is utilizable.

## Simulation results

**Figure 2:**
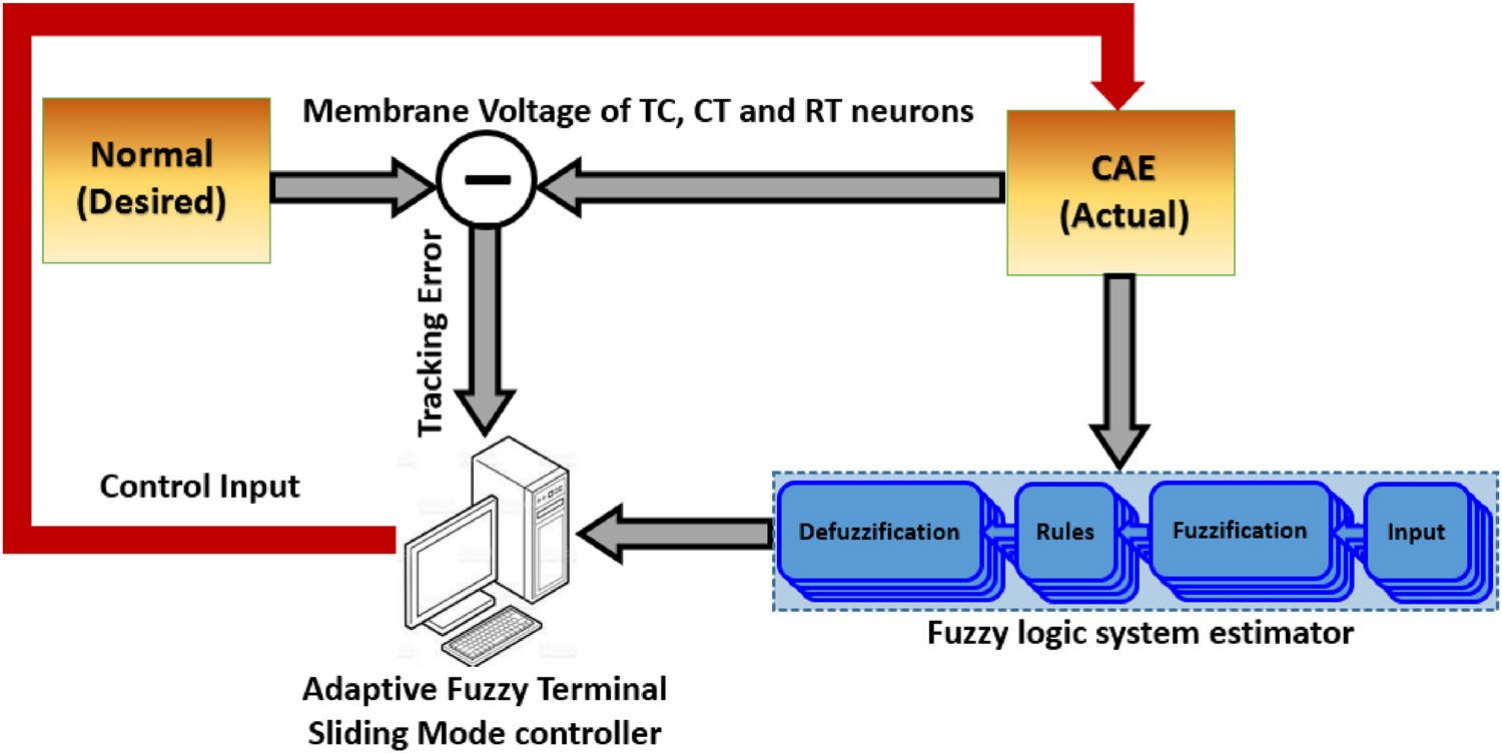
Schematic of the closed-loop control system for elimination of oscillatory spiking pattern through adaptive DBS using AFTSMC.

The simulation results of the CAE and normal states of the spiking patterns of TC, CT, and RT neurons with the tracking results of the AFTSMC for suppression of the oscillatory pattern of the CAE are provided in this section. The schematic of the closed-loop control system through adaptive DBS using AFTSMC is indicated in Fig. 2. To implement the controller, 15 fuzzy estimators are used to approximate the nonlinear dynamics of the plant embedded in the control input. The simulation is performed on the desktop computer (containing an Intel (R) Core (TM) i7-9700K @ 3.60 GHz CPU and 32 GB DDR4 RAM) in MATLAB R2020a Simulink (64-bit) under Windows 10. The sampling period of 0.001 ms is used for updating the adaptive values of the FLS estimator and the controller. The mean running time of the closed-loop system for the total period of 2000 ms simulation is about 25 min.

**Figure 3:**
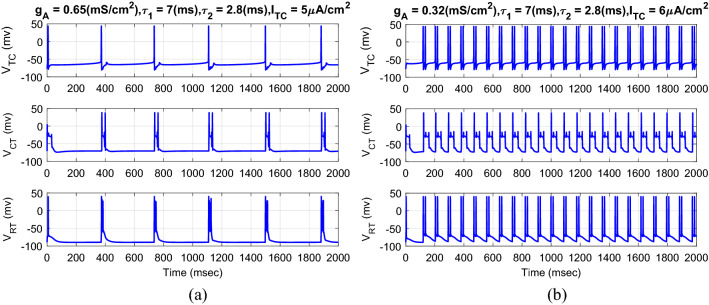
Typical results of the membrane voltage of TC, CT, and RT neurons with the different values of external current of TC neuron and maximal conductance from RT neuron to TC neuron for normal (**a**) and CAE (**b**) states.

Figure 3 shows the typical results of the membrane voltage of TC, CT, and RT neurons in response to the external current of TC neuron for normal and CAE states with the different values of maximal conductance from RT neuron to TC neuron. If the external current of the TC neuron increased from 5 to 6 μA cm^−2^ with the decrement of the strength of the maximal conductance from the RT neuron to the TC neuron from 0.65 to 0.32 mS cm^−2^, the rhythm of the neurons changes from bursting or single spiking to tonic spiking. Indeed, the complexity of the dynamics of the neurons is dependent on the values of the external TC current and the maximal conductance from the RT neuron to the TC neuron. The smaller selection of the maximal conductance, the faster rhythm of the neurons in the CAE state. To calculate the tracking error, the desired trajectory is the membrane voltage of the TC, CT, and RT neurons in the normal state (Fig. 3a). The goal of the AFTSMC is to automatically generate the continuous controlled current (DBS pulses) to return the oscillator spiking patterns of the three neurons to the normal bursting patterns in the presence of uncertainty and disturbance current.

### Adaptive fuzzy terminal sliding mode control of DBS pulses

**Figure 4:**
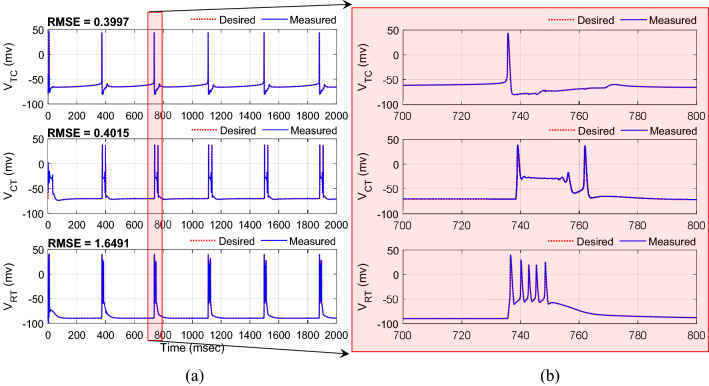
(**a**) Typical results of the tracking using AFTSMC. (**b**) Zoom-in version of (**a**) between 700 and 800 ms.

**Figure 5:**
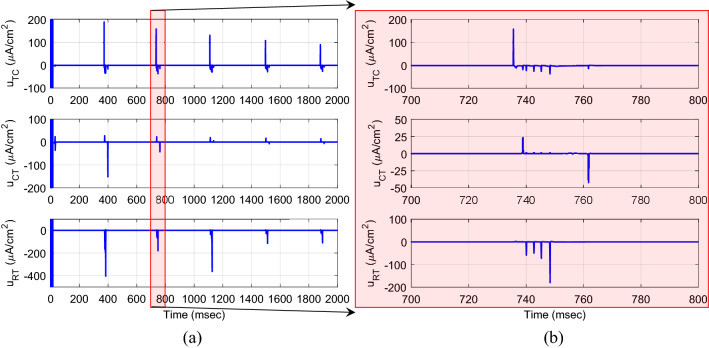
(**a**) Typical results of the control effort using AFTSMC. (**b**) Zoom-in version of (**a**) between 700 and 800 ms.

**Figure 6:**
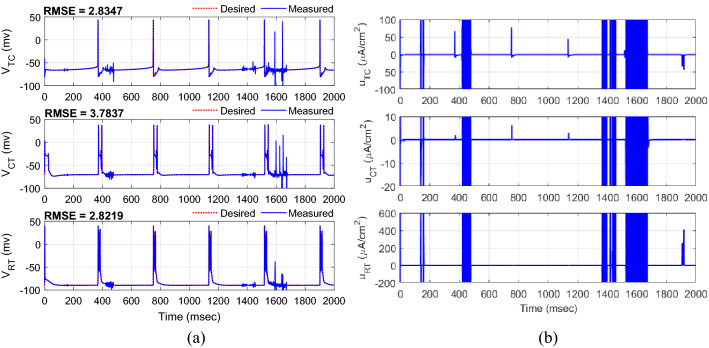
(**a**) Typical results of the tracking using AFSMC. (**b**) Control input.

In this part, the results of the closed-loop tracking of the membrane voltages of the CAE state using the proposed AFTSMC are presented. To evaluate the accuracy of the tracking, the root mean square of the tracking error (RMSE) is calculated in the following form:

31 $$RMSE = \sqrt {\frac{1}{N}\sum\limits_{i = 1}^{N} {\left| {x_{j} (i) - x_{j,d} (i)} \right|^{2} } } ,j \in \left\{ {TC,CT,RT} \right\}$$

where $x_{j}$ and $x_{j,d}$ are the membrane voltage of each neuron for CAE and normal states, respectively and *N* is the number of samples in the total time of the simulation. The input of the fuzzy estimators is the membrane voltage of the TC, CT, and RT neurons and for each voltage five Gaussian-type membership functions were defined in the following:

32 $$\mu_{{A_{j}^{i} }} = e^{{ - \frac{1}{2}\left( {\frac{{x_{j} - c_{j}^{i} }}{{\delta_{j} }}} \right)^{2} }} , \,\, i = 1,\ldots,5 \quad j = 1,\ldots,3$$

where $c_{1}^{1} = c_{2}^{1} = c_{3}^{1} = - 60$, $c_{1}^{2} = c_{2}^{2} = c_{3}^{2} = - 40$, $c_{1}^{3} = c_{2}^{3} = c_{3}^{3} = 0$, $c_{1}^{4} = c_{2}^{4} = c_{3}^{4} = 40$, $c_{1}^{5} = c_{2}^{5} = c_{3}^{5} = 60$ and $\delta_{1} = \delta_{2} = \delta_{3} = 1$ to cover the full possible changes of the states. The initial conditions of the model for the actual and desired states were set in random values between − 20 and − 60 mv and the initial weights of the FLS estimator were set in random values uniformly distributed between 0 and 1. The parameters of the controller are selected with the trial-and-error method to maintain the high accuracy of the tracking (minimum RMSE) and continuous control input (DBS pulses) in the different runs of the closed-loop system, and then kept for further analysis in the presence of uncertainty of the model parameters and external disturbance. The typical results of the tracking for three neurons are indicated in Fig. 4. Excellent tracking with the RMSE of 0.3997, 0.4015, and 1.6491 mv is achieved using the proposed AFTSMC for TC, CT, and RT neurons, respectively. Figure 5 shows the results of the continuous (chattering-free) control input without any excessive control effort. When the membrane voltage of the three neurons is at rest, the controller generates the DBS current with the minimum energy and only in response to the spiking pattern of the desired voltages increases automatically the amplitude of stimulation pulses to a sharp value to rapidly track the peak of the action potential of the desired trajectory. In contrast, due to the discontinuity of the classical AFSMC in the reaching law of the control input (results of Fig. 6) across the sliding surface, the high switching control activity is appeared in the control input and degrades the performance of the tracking and causes the problem in the implementation of the control input in real-time applications.

**Figure 7:**
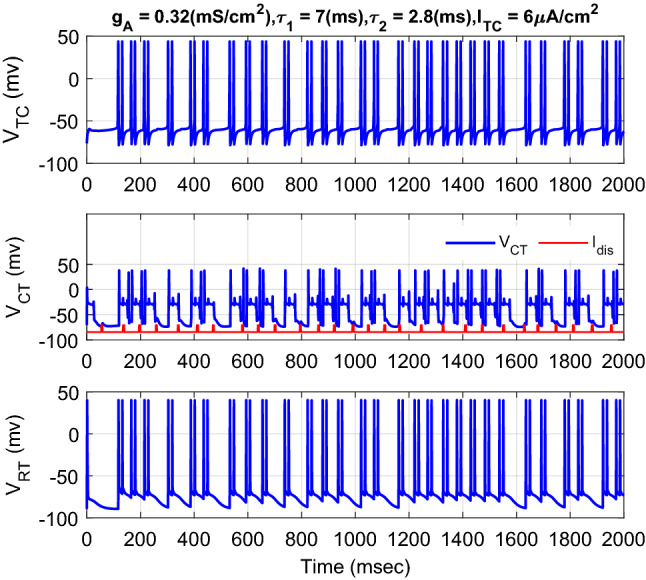
Typical results of the membrane voltage of TC, CT, and RT neurons for CAE state in the presence of the external disturbance current with a gamma distribution with an average rate of 14 Hz applied to CT neuron.

An important issue in controlling the spiking behavior of the CAE model is evaluating the effects of external disturbances on the tracking performance of AFTSMC. The sources of external disturbances may include the un-modeled complexity of the external ionic currents, the unknown synaptic currents from the other neurons in the network applied to the CT, TC, and RT neurons, etc. The proposed controller should robustly handle the effect of these external disturbances without degradation of the tracking performance. To evaluate the ability of the proposed AFTSMC to reject the external disturbance, a non-regular constant current (pulse amplitude, 1.75 μA cm^−2^ and pulse width, 1 ms) was applied to the dynamic equation of TC neuron in (1) for 2000 ms. The pulse is generated based on a gamma distribution with a frequency of 14 Hz. Figure 7 shows the results of the spiking pattern of the membrane voltage of TC, CT, and RT neurons for the CAE state in the presence of the external disturbance current applied to the CT neuron. The results in comparison with the results of Fig. 3(b) indicate that as the random current pulses are applied to the TC neuron, the inter-spike-interval nature of the three neurons changes so that the spiking pattern occurs following the external disturbance current is applied while sometimes as the current pulses are suddenly exerted, the spiking behavior is not observed or is delayed.

**Figure 8:**
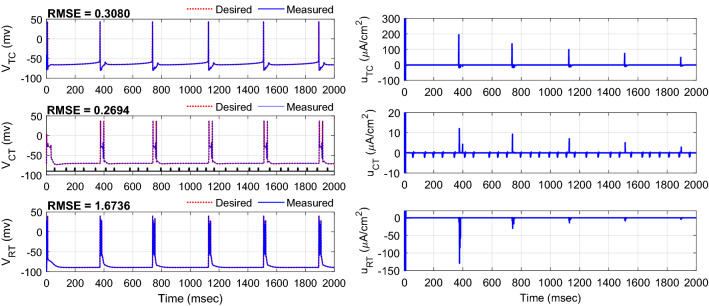
Typical results of the tracking using AFTSMC in the presence of external disturbance current applied to CT neuron.

**Figure 9:**
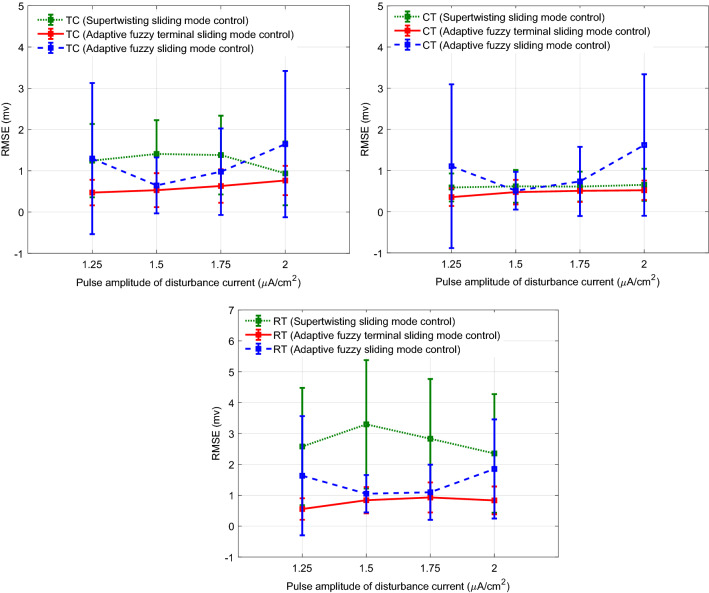
Results of the mean RMSE (± one standard deviation) obtained over 15 trials of the simulation for control of the CAE state using AFTSMC in comparison with the results of AFSMC and STSMC under non-regular external disturbance current applied to CT neuron with different pulse amplitude.

Figure 8 shows the typical results of the tracking under the disturbance current of the CT neuron with a pulse amplitude of 1.25 μA cm^−2^. The RMSE for TC, CT, and RT neurons are 0.3080, 0.2694, and 1.6736 mv, respectively. The controller could effectively adjust the control signal of the TC neuron to reject the effect of the disturbance current applied to the CT neuron. Results of the mean RMSE (± one standard deviation) obtained over 15 trials of the simulation for control of the CAE state in the presence of non-regular external disturbance current applied to CT neuron using AFTSMC in comparison with the AFSMC and super-twisting sliding mode control (STSMC)^70^ are depicted in Fig. 9. The initial conditions of the gating variables of ionic channels and membrane voltage of TC, CT, and RT neurons, the desired pattern of the membrane voltages, the initial weights of the FLS estimators, and the onset of the pulses in the external current applied to the TC neuron were set in random values and changed subsequently from one trial to another trial of the simulation. The mean RMSEs using AFTSMC for the TC neuron with the pulse amplitude of 1.25, 1.5, 1.75, and 2 μA cm^−2^ are 0.47, 0.52, 0.63, and 0.76 mv, respectively, for the CT neuron are 0.35, 0.47, 0.50, and 0.52 mv, respectively, and for the RT neuron are 0.55, 0.83, 0.92, and 0.83 mv, respectively.

**Figure 10:**
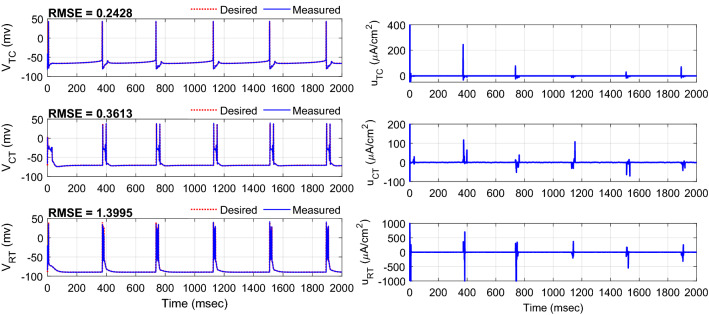
Typical results of the tracking using AFTSMC over 25% uncertainty about the nominal values of maximal conductance and reverse potential of the ionic currents of the three neurons.

**Figure 11:**
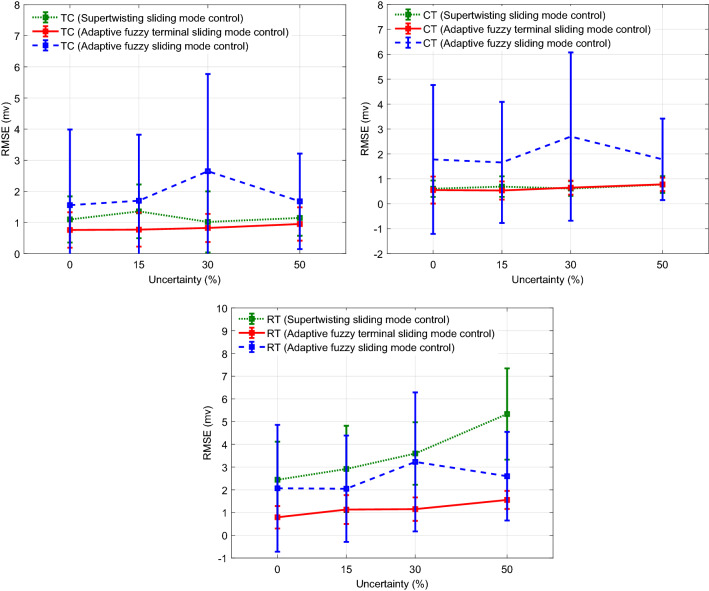
Results of the mean RMSE (± one standard deviation) obtained over 15 trials of the simulation for control of the CAE state using AFTSMC in comparison with the results of AFSMC and STSMC under time-variation of maximal conductance and reverse potential of the ionic currents of three neurons.

To evaluate the robustness of the controller to handle the effect of the time-varying uncertainty of the system parameters, the values of maximal conductance and reverse potential of the ionic currents of three neurons were changed randomly over 0–50% range about the nominal values (last column of the Tables S1, S2, and S3 of Appendix A of the Supplementary Materials) over the total time of the simulation. The variation of each parameter was obtained by passing the uniform distribution sequence with an appropriate selection of its parameters (minimum and maximum) to the second-order low-pass filter with the natural frequency of 0.05 Hz and adding the constant nominal value of the parameter. Figure 10 shows the tracking results and control pulses generated with the proposed controller over 25% uncertainty. The RMSEs over one trial of the simulation are 0.2428, 0.3613, and 1.3995 mv, respectively for TC, CT, and RT neurons. The interesting observation is the automatic generation of positive and negative amplitudes of DBS pulses for tracking control of the RT neuron. In contrast, the amplitude of the DBS pulses generated with the controller in the results of Figs. 5 and 8 was only in the negative range. Figure 11 shows the results of the mean RMSE (± one standard deviation) obtained over 15 trials of the simulation without uncertainty in comparison with random variation of 15, 30, and 50% uncertainty of the maximal conductance and reverse potential of the ionic currents of three neurons in each trial of the simulation through AFTSMC in comparison with the results of AFSMC and STSMC. The mean RMSEs using AFTSMC for the TC neuron without the uncertainty and over 15, 30, and 50% uncertainty are 0.75, 0.77, 0.82, and 0.95 mv, respectively, for the CT neuron are 0.54, 0.53, 0.64, and 0.77 mv, and for the RT neuron are 0.79, 1.13, 1.15, and 1.55 mv, respectively.

## Discussion and conclusions

This paper developed a three-input (continuous DBS pulses), three-output (membrane voltage of neuron) model-based controller using robust AFTSMC to suppress the spiking pattern in a CAE dynamical model. The proposed model consists of cortical, thalamic relay, and reticular nuclei neurons. Although in the previous work^48^, a finite-time fractional order SMC in combination with RBFNN was used to control the epilepsy seizures in a dynamical SWD model, prior knowledge of the model dynamics was required to design the DBS pulses. In contrast, in the current paper, the unknown nonlinear dynamics of the current and delayed states of the model used in the control input were estimated with the adaptive fuzzy controller. A critical issue in controlling the nonlinear dynamical systems in the simulations of brain networks is evaluating the effects of external disturbances and uncertainties on the tracking performance of the closed-loop system. The sources of external disturbances and uncertainties may include the un-modeled complexity of the external ionic currents, the subject-to-subject variability of the parameters, the unknown dynamics (interaction between the nuclei, the unknown different threshold firing levels of neurons in the population), etc. In the current study, the upper bounds of the external disturbance and minimum estimation errors were updated online with adaptive laws without any offline tuning phase. Although increasing the effect of the external disturbance or uncertainty degraded the tracking performance, the results of Figs. 9 and 11 demonstrated the efficiency of the proposed controller in handling the effects of the external disturbance and uncertainty of the model parameters in comparison with the AFSMC and STSMC. Moreover, according to the results of Figs. 8 and 10, the control signal (pulse amplitude) required for the proposed AFTSMC increased sharply in a normal range only when the spiking pattern of the three neurons was generated. Nevertheless, the DBS pulses showed very stable values without any extra activity in comparison with the results of Fig. 6(b) using AFSMC when the pattern of the membrane voltage was in the rest state. But, designing the automatic control pulses using the canonical MIMO nonlinear model is a significant challenge due to the singularity of the estimated matrix gain of the control input during its inverse calculation. To address this limitation in the current study, the regularized form of the estimated matrix with a proper selection of the parameter *ε*_0_ was used. Another challenge facing the MIMO structure of the controlled system is the adjustment of several control parameters to achieve high tracking accuracy and robustness in the presence of external disturbance and uncertainty. To reduce the complexity of the centralized MIMO closed-loop controller with high calculation and easily select the control parameters, a decentralized form of the controller may be an alternative practical approach for the simple implementation of the controller in real DBS systems. The superiority of the decentralized structure is that the MIMO dynamical model is converted to the separated single-input single-output (SISO) subsystems with each controller acts solely on its subsystem, and the interactions between the subsystems are approximated with the adaptive fuzzy controller embedded in each subsystem. The performance of the decentralized closed-loop DBS using AFTSMC should be further investigated and constitutes future research.

In the current study, the structure of the MIMO-controlled system was designed based on the highly nonlinear (conductance-based Hodgkin-Huxley type neuron model) CAE dynamical system, and the adaptive fuzzy controller was recruited to estimate the unknown dynamics of the plant. One important issue that should be considered in a real closed-loop DBS system is the speed of the control algorithm to meet the real-time application. Regarding the computational complexity of the proposed control algorithm, with the sampling period of 0.001 ms, when the algorithm was executed on the CPU the mean run time of the closed-loop system for the total time of the simulation (2000 ms) was about 25 min. It is worth noting that a CPU sequentially processes the data. But, in real-time applications of closed-loop DBS, the control algorithm should be implemented on an FPGA (Field-Programmable Gate Array) which is an integrated circuit with so many parallel hardware resources. Implementation of the control algorithm on the FPGA benefits from parallel data processing and this leads to a great reduction in the run time of the proposed procedure, which may be addressed in future studies.

In a real DBS system, the monophasic (anodic or cathodic) and biphasic (with and without inter-pulse delay between symmetric and asymmetric anodic and cathodic phases) waveforms^76^ may be generally used to deliver the stimuli pulses. But, to satisfy neural tissue safety, the pulse trains of charge-balanced biphasic currents are delivered into the neural tissues^77,78^ and the stimulation parameters (pulse amplitude, pulse width, and frequency) can be adjusted individually or simultaneously with the control algorithms. Short pulse width reduces the damage risk of the neural tissue, and the increment of the pulse amplitude depends on the spatial relationship between the tip of the electrode and the target neural fibers. The high frequency of the stimulation can directly affect the average power consumption of the DBS hardware and decreases its battery life. Another problem is the spread of the electric field due to the location and geometry of the tip of the electrode during selective stimulation of the special neural tissues. The admissible amplitude or width of the stimuli pulses should be appropriately adjusted to focally stimulate the target brain nucleus with the lowest effect on the neighboring non-target tissues. In the current study, the control gain of the model in (3) was considered as a 3 × 3 nonlinear matrix and 9 fuzzy estimators were used to estimate the effect of the three control inputs on the dynamics of each neuron. Thus, we believe that the proposed control scheme can automatically adjust the pulse amplitude or pulse width of the DBS pulses with the electrodes implanted in the region of appropriate neural fibers to suppress the seizure in a real-time closed-loop DBS system.

## Limitations

In the closed-loop stimulation approaches, different physiological signals are used in controlling the neurostimulator output. These data sources include LFP^79^, ECoG^80^, electromyogram (EMG)^80^ and kinematic (acceleration)^81^. Selection of the appropriate biological signals depends on resolution, invasiveness, and relevance to the patient's symptoms. In previous closed-loop DBS methods, the recorded biosignal time series were not directly applied as the input to the controller. In fact, the researchers processed the recorded signals and extracted different features to control the stimulator output. Synchrony of the neural oscillations^48^ and sub-band power of the brain signals^82^ are examples of the considered features in DBS which are relevant to the disease symptoms. But, in the proposed closed-loop DBS technique, the recorded membrane voltage directly drives the controller without any additional processing stage. In other words, in the proposed method, the control signal varies in a continuous manner such that the output of the plant tracks the desired membrane voltage. Since the stable intracellular recording of the action potentials requires periodic calibration of the experimental setup, the long-term clinical application of this approach is limited. To eliminate the limitation of the tracking-based controller and make it suitable for clinical applications, the time series tracking error should be substituted by an appropriate feature tracking error. A candidate feature can be the synchrony of the LFP signals of the brain nuclei. Another limitation of the proposed control scheme in designing the control input is the delay of the model states. In this paper, to estimate the nonlinear dynamics of the delayed states in the input of the fuzzy estimator it was assumed that the value of the delay is known. To hardware implementation of the proposed control algorithm in a real-time closed-loop DBS system, since the delay in the nonlinear dynamics of the system is unknown it should be approximated online using the time-delay estimation (TDE) methods^83,84^ such that the stability of the closed-loop system including TDE algorithm is guaranteed.

## Supplementary Information

Supplementary Information.

## Data Availability

No datasets were used in the current study and all data needed for the simulation are included in this published article and its supplementary materials file. The MATLAB codes used for the generation of the results are available from the corresponding author on reasonable request.
