# Supplementary material for: Suppression of seizure in childhood absence epilepsy using robust control of deep brain stimulation: a simulation study
Source: Sci Rep. 2023 Jan 10;13:461. doi: 10.1038/s41598-023-27527-1 (PMC9832016; doi:10.1038/s41598-023-27527-1)
Supplement: Supplementary file 1 — Supplementary Information. [file 41598_2023_27527_MOESM1_ESM.docx]

**Supplementary Materials for “Suppression of seizure in childhood absence epilepsy using robust control of deep brain stimulation: A simulation study”**

Ehsan Rouhani ^1*^, Ehsan Jafari ^2^, Amir Akhavan ^1^

^1^ Department of Electrical and Computer Engineering, Isfahan University of Technology, Isfahan 84156-83111, Iran.

^2^ CNRS UMR 5672, Ecole Normale Supérieure de Lyon, 46 allée d’Italie, 69007, Lyon, France.

**Appendix A. Details of the ionic currents of the model**

In this Appendix, the details of ionic membrane currents with their dynamical equations and parameters are provided. The leak current equation for TC, CT, and RT neurons is as follows:

$I_{L}=G_{L}\left(V_{i}-E_{L}\right)$, (S.1)

where $V_{i},i\in\left\{TC,CT,RT\right\}$is the membrane voltage, $G_{L}$is the maximal conductance, and $E_{L}$is the reversal potential. The transient voltage-gated current of Na^+^ ions and the dynamic equations of the gating variables of Na^+^ ionic channels are as follows:

$I_{Na}=G_{Na}m_{Na}^{3}h_{Na}\left(V_{i}-E_{Na}\right),i\in\left\{TC,CT,RT\right\}$ (S.2)

$\frac{dm_{Na}}{dt}=\alpha_{m_{Na}}(1-m_{Na})-\beta_{m_{Na}}m_{Na}$ (S.3)

$\frac{dh_{Na}}{dt}=\alpha_{h_{Na}}(1-h_{Na})-\beta_{h_{Na}}h_{Na}$, (S.4)

where$m_{Na}$ and$h_{Na}$are the gating variable of Na^+^ ionic channels and the parameters $\alpha_{m_{Na}},\beta_{m_{Na}},\alpha_{h_{Na}},\beta_{h_{Na}}$ are summarized in Tables S1, S2, and S3 for TC, CT, and RT neurons, respectively. The transient voltage-gated current of K^+^ ions and its dynamic equation for the gating variable are as follows:

$I_{K}=G_{K}m_{K}^{4}\left(V_{TC}-E_{K}\right)$ (S.5)

$\frac{dm_{K}}{dt}=\alpha_{m_{K}}(1-m_{K})-\beta_{m_{K}}m_{K}$, (S.6)

where$m_{K}$is the gating variable of K^+^ ionic channels and the parameters $\alpha_{m_{K}}$and$\beta_{m_{K}}$are summarized in Tables S1, S2, and S3 for TC, CT, and RT neurons, respectively. The current equation of the low-threshold current of Ca^++^ ions and the dynamics of the gating variables for TC neuron are as follows:

$I_{T}=-G_{T}m_{T}^{3}h_{T}\left(V_{TC}-E_{T}\right)$ (S.7)

$\frac{dm_{T}}{dt}=\frac{m_{\infty_{T}}-m_{T}}{\tau_{m_{T}}}$ (S.8)

$\frac{dh_{T}}{dt}=\alpha_{1}(1-h_{T}-d_{T}-Kh_{T})$ (S.9)

$\frac{d}{dt}d_{T}=\alpha_{2}(K\left(1-h_{T}-d_{T}\right)-d_{T})$, (S.10)

where$m_{T}$,$h_{T}$, and$d_{T}$are the gating variables of Ca^++^ ionic channels, and the parameters $m_{\infty_{T}},\tau_{m_{T}},\alpha_{1},\alpha_{2},K$ are summarized in Table S1.The equation of the mixed Na^+^-K^+^ current activated by hyperpolarization for the TC neuron and its dynamic equations for the gating variables are as follows:

$I_{h}=G_{h}S_{1}F_{1}\left(V_{TC}-E_{h}\right)$ (S.11)

$\frac{dS_{1}}{dt}=\frac{h_{\infty_{s}}-S_{1}}{\tau_{S}}$ (S.12)

$\frac{dF_{1}}{dt}=\frac{h_{\infty_{s}}-F_{1}}{\tau_{F}}$, (S.13)

where$S_{1}$and $F_{1}$are the gating variables of mixed Na^+^-K^+^ ionic channels and the parameters $h_{\infty_{s}},\tau_{S},\tau_{F}$ are summarized in Table S1. The current equation of the slow current of K^+^ ions and the dynamics of the gating variables for TC neuron are provided as follows:

$I_{K_{2}}=G_{K_{2}}m_{2}\left(0.6h_{1}+0.4h_{2}\right)\left(V_{TC}-E_{K}\right)$ (S.14)

$\frac{dm_{2}}{dt}=\frac{m_{2_{\infty}}-m_{2}}{\tau_{m_{2}}}$ (S.15)$\frac{dh_{1}}{dt}=\frac{h_{2_{\infty}}-h_{1}}{\tau_{h_{1}}}$ (S.16)

$\frac{dh_{2}}{dt}=\frac{h_{2_{\infty}}-h_{2}}{\tau_{h_{2}}}$, (S.17)

where$h_{1}$,$h_{2}$, and$m_{2}$are the gating variables of slow K^+^ ionic channels, and the parameters $m_{2_{\infty}},h_{2_{\infty}},\tau_{m_{2}},\tau_{h_{2}}$ are summarized in Table S1. The current equation of the depolarization-activated current of K^+^ ions and its gating variable dynamic for the CT neuron are as follows:

$I_{M}=-G_{M}m_{M}\left(V_{CT}-E_{M}\right)$ (S.18)

$\frac{dm_{M}}{dt}=\frac{m_{\infty_{M}}-m_{M}}{\tau_{m_{M}}}$, (S.19)

where$m_{M}$is the gating variable of K^+^ ionic channels and the parameters $m_{\infty_{M}},\tau_{m_{M}}$ are summarized in Table S2. The current equation of the low-threshold current of Ca^++^ ions and their dynamic equations of the gating variables for the RT neuron are defined as follows:

$I_{TS}=G_{Ca}m_{TS}^{2}h_{TS}\left(V_{RT}-E_{Ca}\right)$ (S.20)

$\frac{dm_{TS}}{dt}=\frac{m_{\infty_{TS}}-m_{TS}}{\tau_{m_{TS}}}$ (S.21)

$\frac{dh_{TS}}{dt}=\frac{h_{\infty_{TS}}-h_{TS}}{\tau_{h_{TS}}}$, (S.22)

where$m_{TS}$and$h_{TS}$are the gating variable of Ca^++^ ionic channels and the parameters $m_{\infty_{TS}},h_{\infty_{TS}},\tau_{m_{TS}},\tau_{h_{TS}}$ are summarized in Table S3. The equations of the gating variables and the parameters values for TC, CT, and RT neurons are summarized in Tables S1, S2, and S3, respectively.

**Table S1.** Current equations and model parameters for the TC neuron.

| Current | Gating variables | Parameters values |
| --- | --- | --- |
| $I_{L}$ | - | $\begin{matrix} G_{L}=0.01 \\ E_{L}=-70 \end{matrix}$ |
| $I_{Na}$ | $\alpha_{m_{Na}}=0.32\left(13-\left(V_{TC}+64\right)\right)/\left(e^{\left(\left(13-\max\left(\left(V_{TC}+64\right),-60\right)\right)/4\right)}-1\right)$ $\beta_{m_{Na}}=0.28\left(\left(V_{TC}+64\right)-40\right)/\left(e^{\left(\left(\min\left(\left(V_{TC}+64\right),70\right)-40\right)/5\right)}-1\right)$ $\alpha_{h_{Na}}=0.128e^{\left(\left(17-\max\left(\left(V_{TC}+64\right),-80\right)\right)/18\right)}$ $\beta_{h_{Na}}=4/\left(e^{\left(\left(40-\max\left(\left(V_{TC}+64\right),-80\right)\right)/5\right)}+1\right)$ | $\begin{matrix} G_{Na}=90 \\ E_{Na}=45 \end{matrix}$ |
| $I_{K}$ | $\alpha_{m_{K}}=0.032\left(15-\left(V_{TC}+64\right)\right)/\left(e^{\left(\left(15-\max\left(\left(V_{TC}+64\right),-80\right)\right)/5\right)}-1\right)$ $\beta_{m_{K}}=0.5e^{\left(\left(10-\max\left(\left(V_{TC}+64\right),-80\right)\right)/40\right)}$ | $\begin{matrix} G_{K}=10 \\ E_{K}=-90 \end{matrix}$ |
| $I_{T}$ | $m_{\infty_{T}}=1/\left(1+e^{\left(\left(-\max\left(V_{TC},-80\right)-65\right)/7.8\right)}\right)$ $\tau_{m_{T}}=0.15m_{\infty_{T}}\left(1.7+e^{\left(\left(-\max\left(V_{TC},-80\right)-30.8\right)/13.5\right)}\right)$ $\alpha_{1}=e^{\left(\left(-\max\left(V_{TC},-80\right)-162.3\right)/17.8\right)}/0.26$ $K=\sqrt{0.25+e^{\left(\left(\min\left(V_{TC},50\right)+85.5\right)/6.3\right)}}-0.5$ $\tau_{2}=62.4/\left(1+e^{\left(\left(\min\left(V_{TC},80\right)+39.4\right)/30\right)}\right)$ $\alpha_{2}=1/\left(\tau_{2}\left(K+1\right)\right)$ | $\begin{matrix} G_{T}=2 \\ E_{K}=120 \end{matrix}$ |
| $I_{h}$ | $h_{\infty_{s}}=1/\left(1+e^{\left(\left(\min\left(V_{TC},60\right)+68.9\right)/6.5\right)}\right)$ $\tau_{s}=\max\left(e^{\left(\left(\min\left(V_{TC},60\right)+183.26\right)/15.24\right)},0.01\right)$ $\tau_{F}=\max\left(\left(e^{\left(\left(\min\left(V_{TC},60\right)+158.6\right)/11.2\right)}/\left(1+e^{\left(\left(\min\left(V_{TC},50\right)+75\right)/5.5\right)}\right)\right),0.01\right)$ | $\begin{matrix} G_{h}=0.02 \\ E_{h}=-43 \end{matrix}$ |
| $I_{K_{2}}$ | $m_{2_{\infty}}=1/\left(1+e^{\left(-\left(V_{TC}+43\right)/17\right)}\right)$ $\tau_{m_{2}}=2.86+0.29/\left(e^{\left(\left(V_{TC}-81\right)/25.6\right)}+e^{\left(-\left(V_{TC}+132\right)/25.6\right)}\right)$ $h_{2_{\infty}}=1/\left(1+e^{\left(\left(V_{TC}+58\right)/10.6\right)}\right)$ $\tau_{h_{1}}=34.65+0.29/\left(e^{\left(\left(V_{TC}-1329\right)/200\right)}+e^{\left(-\left(V_{TC}+130\right)/71\right)}\right)$ $\tau_{h_{2}}=\left\{\begin{matrix} \tau_{h_{1}}\text{ }if\text{ }V_{TC}\geq-70 \\ 2570\text{ }if\text{ }V_{TC}<-70 \end{matrix}\right.$ | $G_{K_{2}}=5E-5$ |

**Table S2.** Current equations and model parameters for the CT neuron.

| Current | Gating variables | Parameters |
| --- | --- | --- |
| $I_{L}$ | - | $\begin{matrix} G_{L}=0.1 \\ E_{L}=-70 \end{matrix}$ |
| $I_{Na}$ | $\alpha_{m_{Na}}=0.32\left(13-\left(V_{CT}+64\right)\right)/\left(e^{\left(\left(13-\max\left(\left(V_{CT}+64\right),-60\right)\right)/4\right)}-1\right)$ $\beta_{m_{Na}}=0.28\left(\left(V_{CT}+64\right)-40\right)/\left(e^{\left(\left(\min\left(\left(V_{CT}+64\right),70\right)-40\right)/5\right)}-1\right)$ $\alpha_{h_{Na}}=0.128e^{\left(\left(17-\max\left(\left(V_{CT}+64\right),-80\right)\right)/18\right)}$ $\beta_{h_{Na}}=4/\left(e^{\left(\left(40-\max\left(\left(V_{CT}+64\right),-80\right)\right)/5\right)}+1\right)$ | $\begin{matrix} G_{Na}=50 \\ E_{Na}=45 \end{matrix}$ |
| $I_{K}$ | $\alpha_{m_{K}}=0.032\left(15-\left(V_{CT}+64\right)\right)/\left(e^{\left(\left(15-\max\left(\left(V_{CT}+64\right),-80\right)\right)/5\right)}-1\right)$ $\beta_{m_{K}}=0.5e^{\left(\left(10-\max\left(\left(V_{CT}+64\right),-80\right)\right)/40\right)}$ | $\begin{matrix} G_{K}=5 \\ E_{K}=-90 \end{matrix}$ |
| $I_{M}$ | $m_{\infty_{M}}=1/\left(1+e^{\left(-\left(V_{CT}+35\right)/10\right)}\right)$ $\tau_{M}=1000/\left(3.3\left(e^{\left(\left(V_{CT}+35\right)/20\right)}+e^{\left(-\left(V_{CT}+35\right)/20\right)}\right)\right)$ | $\begin{matrix} G_{M}=0.07 \\ E_{M}=-100 \end{matrix}$ |

**Table S3.** Current equations and model parameters for the RT neuron.

| Current | Gating variables | Parameters |
| --- | --- | --- |
| $I_{L}$ | - | $\begin{matrix} G_{L}=0.05 \\ E_{L}=-90 \end{matrix}$ |
| $I_{Na}$ | $\alpha_{m_{Na}}=0.32\left(13-\left(V_{RT}+64\right)\right)/\left(e^{\left(\left(13-\max\left(\left(V_{RT}+64\right),-60\right)\right)/4\right)}-1\right)$ $\beta_{m_{Na}}=0.28\left(\left(V_{RT}+64\right)-40\right)/\left(e^{\left(\left(\min\left(\left(V_{RT}+64\right),70\right)-40\right)/5\right)}-1\right)$ $\alpha_{h_{Na}}=0.128e^{\left(\left(17-\max\left(\left(V_{RT}+64\right),-80\right)\right)/18\right)}$ $\beta_{h_{Na}}=4/\left(e^{\left(\left(40-\max\left(\left(V_{RT}+64\right),-80\right)\right)/5\right)}+1\right)$ | $\begin{matrix} G_{Na}=200 \\ E_{Na}=45 \end{matrix}$ |
| $I_{K}$ | $\alpha_{m_{K}}=0.032\left(15-\left(V_{RT}+64\right)\right)/\left(e^{\left(\left(15-\max\left(\left(V_{RT}+64\right),-80\right)\right)/5\right)}-1\right)$ $\beta_{m_{K}}=0.5e^{\left(\left(10-\max\left(\left(V_{RT}+64\right),-80\right)\right)/40\right)}$ | $\begin{matrix} G_{K}=20 \\ E_{K}=-80 \end{matrix}$ |
| $I_{TS}$ | $m_{\infty_{TS}}=1/\left(1+e^{\left(-\left(V_{RT}+52\right)/7.4\right)}\right)$ $\tau_{M_{TS}}=1+0.33/\left(e^{\left(\left(V_{RT}+27\right)/10\right)}+e^{\left(-\left(V_{RT}+102\right)/15\right)}\right)$ $h_{\infty_{TS}}=1/\left(1+e^{\left(\left(V_{RT}+80\right)/5\right)}\right)$ $\tau_{h_{TS}}=28.3+0.33/\left(e^{\left(\left(V_{RT}+48\right)/4\right)}+e^{\left(-\left(V_{RT}+407\right)/50\right)}\right)$ | $\begin{matrix} G_{Ca}=3 \\ E_{Ca}=120 \end{matrix}$ |

**Appendix B. Fuzzy logic estimator**

In this Appendix, the details of the fuzzy logic estimator are provided. To estimate the functions $\mathbf{f}(\mathbf{x},t)$, $\mathbf{f}_{\tau}(\mathbf{x}_{\tau},t)$and $\mathbf{G}(\mathbf{x},t)$, $m(m+2)$ fuzzy rules are designed to map the input vector $\mathbf{x}=[x_{1},x_{2},...x_{n}]^{T}\in R^{n}$ to an output$y\in R$. If we use the singleton fuzzifier, product-inference engine, and center-average defuzzifier, the output of the FLS is given with the following function:

$y=\frac{\sum_{i=1}^{n_{r}} \tilde{y}^{i}\left(\prod_{j=1}^{n} \mu_{A_{j}^{i}}(x_{j})\right)}{\sum_{i=1}^{n_{r}} \left(\prod_{j=1}^{n} \mu_{A_{j}^{i}}(x_{j})\right)}=\mathbf{\psi}^{T}\mathbf{\zeta}(\mathbf{x})$. (S.23)

In (S.23), $n_{r}$is the total number of fuzzy rules, $\tilde{y}^{i}$ is the output at a point which $\mu_{B^{i}}(\tilde{y}^{i})$reaches its maximum value, and $\mu_{A_{j}^{i}}(x_{j})$ is the membership function of the fuzzy set $A_{j}^{i}$and is specified by the Gaussian function. $\mathbf{\psi}=\left[\tilde{y}^{1},\tilde{y}^{2}\ldots,\tilde{y}^{n_{r}}\right]^{T}$ is an adjustable vector group of the parameters and $\mathbf{\zeta}=\left[\zeta^{1},\zeta^{2},\ldots\zeta^{n_{r}}\right]^{T}$ is a fuzzy basis vector function. By using the fuzzy approximator in the form of (S.23), the estimation of nonlinear functions $\mathbf{f}(\mathbf{x},t)$, $\mathbf{f}_{\tau}(\mathbf{x}_{\tau},t)$, and $\mathbf{G}(\mathbf{x},t)$ are written as follows:

$\begin{matrix} \hat{f}_{i}(\mathbf{x},\psi_{_{f_{_{i}}}}^{t})=\psi_{_{f_{_{i}}}}^{T}\zeta_{f_{_{i}}}\mathbf{(x)}\text{, }i=1, \ldots, m \\ \hat{f}_{\tau_{i}}(\mathbf{x}_{\tau},\psi_{_{f_{_{\tau_{i}}}}}^{t})=\psi_{_{f_{_{\tau_{i}}}}}^{T}\zeta_{f_{_{\tau_{i}}}}\mathbf{(x}_{\tau}\mathbf{)}\text{, }i=1, \ldots, m \end{matrix}$ (S.24)

$\hat{g}_{ij}(\mathbf{x},\psi_{_{g_{ij}}}^{t})=\psi_{_{g_{ij}}}^{T}\zeta_{g_{ij}}\mathbf{(x)}\text{, }i, j=1, \ldots, m$ (S.25)

where $\psi_{_{f_{_{i}}}}^{t},\psi_{_{f_{_{\tau_{i}}}}}^{t}$ and $\psi_{_{g_{ij}}}^{t}$are the corresponding adaptive vectors and $\zeta_{f_{_{i}}}\mathbf{(x)}$, $\zeta_{f_{_{\tau_{i}}}}\mathbf{(x}_{\tau}\mathbf{)}$, and $\zeta_{g_{ij}}\mathbf{(x)}$ denote fuzzy basis vector fixed with the designer. The optimal values $\psi_{_{f_{_{i}}}}^{*},\psi_{_{f_{2_{ij}}}}^{*}$ and $\psi_{g_{ij}}^{*}$are defined as

$\begin{matrix} \psi_{_{f_{i}}}^{*}=\arg\min_{\psi_{_{f_{_{i}}}}^{t}} \left\{\sup_{x\in D_{x}} \left|f_{i}(\mathbf{x},t)-\hat{f}_{i}(\mathbf{x},\psi_{f_{i}}^{t})\right|\right\} \\ \psi_{_{f_{\tau_{i}}}}^{*}=\arg\min_{\psi_{f_{\tau_{i}}}^{t}} \left\{\sup_{x\in D_{x}} \left|f_{\tau_{i}}(\mathbf{x}_{\tau},t)-\hat{f}_{\tau_{i}}(\mathbf{x}_{\tau},\psi_{f_{\tau_{i}}}^{t})\right|\right\} \end{matrix}$ (S.26)

$\psi_{g_{ij}}^{*}=\arg\min_{\psi_{_{g_{ij}}}^{t}} \left\{\sup_{x\in D_{x}} \left|g_{ij}(\mathbf{x},t)-\hat{g}_{ij}(\mathbf{x},\psi_{g_{ij}}^{t})\right|\right\}$. (S.27)

These values are artificial constant parameters introduced only for stability proof without the requirement of the controller to their values.

**Appendix C. Proof of the Theorem 2**

In this Appendix, the proof of Theorem 2 is presented. By substituting (24) into (13), it yields

(S.28)

By substituting $\mathbf{u}_{c}(t)$ in (18) into $\hat{\mathbf{G}}(\mathbf{x},\mathbf{\psi}_{g}^{t})\mathbf{u}_{c}(t)$ term of (S.28), we have

(S.29)

By using (19) and (20) we have

$\begin{matrix} \mathbf{f}(\mathbf{x},t)-\hat{\mathbf{f}}(\mathbf{x},\mathbf{\psi}_{f}^{t})=\mathbf{f}^{*}(\mathbf{x},\mathbf{\psi}_{f}^{*})-\hat{\mathbf{f}}(\mathbf{x},\mathbf{\psi}_{f}^{t})+\mathbf{\varepsilon}_{f}(\mathbf{x}) \\ \mathbf{f}_{\tau}(\mathbf{x}_{\tau},t)-\hat{\mathbf{f}}_{\tau}(\mathbf{x}_{\tau},\mathbf{\psi}_{f_{\tau}}^{t})=\mathbf{f}_{\tau}^{*}(\mathbf{x}_{\tau},\mathbf{\psi}_{f_{\tau}}^{*})-\hat{\mathbf{f}}_{\tau}(\mathbf{x}_{\tau},\mathbf{\psi}_{f_{\tau}}^{t})+\mathbf{\varepsilon}_{f_{\tau}}(\mathbf{x}_{\tau}) \end{matrix}$ (S.30)

$\mathbf{G}(\mathbf{x},t)-\hat{\mathbf{G}}(\mathbf{x},\mathbf{\psi}_{g}^{t})=\mathbf{G}^{*}(\mathbf{x},\mathbf{\psi}_{g}^{*})-\hat{\mathbf{G}}(\mathbf{x},\mathbf{\psi}_{g}^{t})+\mathbf{\varepsilon}_{g}(\mathbf{x})$. (S.31)

Substituting (S.30) and (S.31) into (S.29) yields

(S.32)

By multiplying $\mathbf{s}^{T}$both sides of the (S.32), we have

(S.33)

where in (S.33) $\tilde{\psi}_{f_{i}}=\psi_{f_{i}}^{*}-\psi_{f_{i}}^{t},\tilde{\psi}_{f_{\tau_{i}}}=\psi_{f_{\tau_{i}}}^{*}-\psi_{f_{\tau_{i}}}^{t}$ and $\tilde{\psi}_{g_{ij}}=\psi_{g_{ij}}^{*}-\psi_{g_{ij}}^{t}$are the estimation errors of the parameters of FLS.

**Part 1.** Consider the Lyapunov function as follows:

$\begin{matrix} V=\frac{1}{2}\mathbf{s}^{T}\mathbf{s}+\frac{1}{2}\sum_{i=1}^{m} \frac{1}{\kappa_{f_{i}}}\tilde{\psi}_{f_{i}}^{T}\tilde{\psi}_{f_{i}}+\frac{1}{2}\sum_{i=1}^{m} \frac{1}{\kappa_{f_{\tau_{i}}}}\tilde{\psi}_{f_{\tau_{i}}}^{T}\tilde{\psi}_{f_{\tau_{i}}}+\frac{1}{2}\sum_{i=1}^{m} \sum_{j=1}^{m} \frac{1}{\kappa_{g_{ij}}}\tilde{\psi}_{g_{ij}}^{T}\tilde{\psi}_{g_{ij}}+\frac{1}{2}\sum_{i=1}^{m} \frac{1}{\gamma_{0}}\tilde{\varepsilon}_{f_{i}}^{T}\tilde{\varepsilon}_{f_{i}}+ \\ \text{ }\frac{1}{2}\sum_{i=1}^{m} \frac{1}{\gamma_{1}}\tilde{\varepsilon}_{f_{\tau_{i}}}^{T}\tilde{\varepsilon}_{f_{\tau_{i}}}+\frac{1}{2\gamma_{2}}\tilde{\varepsilon}_{g}^{T}\tilde{\varepsilon}_{g}+\frac{1}{2}\sum_{i=1}^{m} \frac{1}{\gamma_{3}}\tilde{d}_{i}^{T}\tilde{d}_{i}+\frac{1}{2\kappa_{0}}ϒ^{2}, \end{matrix}$ (S.34)

where the parameters$\tilde{\mathbf{\varepsilon}}_{f},\tilde{\mathbf{\varepsilon}}_{f_{\tau}},\tilde{\mathbf{\varepsilon}}_{g},$and $\tilde{\mathbf{d}}$are the estimation error of the bound of the parameters$\mathbf{\varepsilon}_{f},\mathbf{\varepsilon}_{f_{\tau}},\mathbf{\varepsilon}_{g},$and $\mathbf{d}$respectively. By differentiating (S.34) with respect to time we have

(S.35)

Substituting (S.33) into (S.35), yields

, (S.36)

where

(S.37)

(S.38)

By substituting the fuzzy adaptation laws (22) and (23) in (S.37), is obtained. Using assumption 1 and equation (25), yields

$\begin{matrix} \mathbf{s}^{T}\mathbf{G}(\mathbf{x},t)\mathbf{u}_{r}(t)\geq\sigma_{0}\left\|\mathbf{s}\right\|^{2}\frac{\left|\mathbf{s}^{T}\right|\text{ }(\hat{\mathbf{\varepsilon}}_{f}+\hat{\mathbf{\varepsilon}}_{f_{\tau}}+\hat{\varepsilon}_{g}\left|\mathbf{u}_{c}\right|+\hat{\mathbf{d}}+\left|\mathbf{u}_{0}\right|)}{\sigma_{0}\left\|\mathbf{s}\right\|^{2}+ϒ}=\left|\mathbf{s}^{T}\right|\text{ }(\hat{\mathbf{\varepsilon}}_{f}+\hat{\mathbf{\varepsilon}}_{f_{\tau}}+\hat{\varepsilon}_{g}\left|\mathbf{u}_{c}\right|+\hat{\mathbf{d}}+\left|\mathbf{u}_{0}\right|)- \\ ϒ\frac{\left|\mathbf{s}^{T}\right|\text{ }(\hat{\mathbf{\varepsilon}}_{f}+\hat{\mathbf{\varepsilon}}_{f_{\tau}}+\hat{\varepsilon}_{g}\left|\mathbf{u}_{c}\right|+\hat{\mathbf{d}}+\left|\mathbf{u}_{0}\right|)}{\sigma_{0}\left\|\mathbf{s}\right\|^{2}+ϒ}. \end{matrix}$ (S.39)

Thus, (S.38) is bounded as follows:

, (S.40)

where$\mathbf{\varepsilon}_{f}^{*}(\mathbf{x})=\tilde{\mathbf{\varepsilon}}_{f}(\mathbf{x})+\hat{\mathbf{\varepsilon}}_{f}(\mathbf{x}),\mathbf{\varepsilon}_{f_{\tau}}^{*}(\mathbf{x})=\tilde{\mathbf{\varepsilon}}_{f_{\tau}}(\mathbf{x})+\hat{\mathbf{\varepsilon}}_{f_{\tau}}(\mathbf{x}),\varepsilon_{g}^{*}(\mathbf{x})=\tilde{\varepsilon}_{g}(\mathbf{x})+\hat{\varepsilon}_{g}(\mathbf{x}),\mathbf{d}^{*}(\mathbf{x})=\tilde{\mathbf{d}}(\mathbf{x})+\hat{\mathbf{d}}(\mathbf{x})$. Thus, using (S.39), the equation (S.40) can be rewritten

(S.41)

By substituting the adaptation law(Eq. (26) in (S.41) we have

(S.42)

Equation (S.36) can be written as

. (S.43)

Thus, all signals in the closed-loop system are bounded.

**Part 2.** Consider a Lyapunov function$V'=0.5\mathbf{s}^{T}\mathbf{s}$. It follows from (S.32) and (S.39) that the derivative of$V'$can be written as

(S.44)

In (S.44) if we define

$\begin{matrix} \left(\mathbf{f}^{*}(\mathbf{x},\mathbf{\psi}_{f}^{*})-\hat{\mathbf{f}}(\mathbf{x},\mathbf{\psi}_{f}^{t})\right)+\left(\mathbf{f}_{\tau}^{*}(\mathbf{x}_{\tau},\mathbf{\psi}_{f_{\tau}}^{*})-\hat{\mathbf{f}}_{\tau}(\mathbf{x}_{\tau},\mathbf{\psi}_{f_{\tau}}^{t})\right)+\left(\mathbf{G}^{*}(\mathbf{x},\mathbf{\psi}_{g}^{*})-\hat{\mathbf{G}}(\mathbf{x},\mathbf{\psi}_{g}^{t})\right)\mathbf{u}_{c}(t) \\ -\left(sign(\mathbf{s}^{T})\cdot\left(\tilde{\mathbf{\varepsilon}}_{f}(\mathbf{x})+\tilde{\mathbf{\varepsilon}}_{f_{\tau}}(\mathbf{x})+\tilde{\mathbf{d}}(\mathbf{x})+\tilde{\varepsilon}_{g}(\mathbf{x})\left|\mathbf{u}_{c}\right|\right)+ϒsign(\mathbf{s}^{T})\cdot\frac{(\hat{\mathbf{\varepsilon}}_{f}+\hat{\mathbf{\varepsilon}}_{f_{\tau}}+\hat{\varepsilon}_{g}\left|\mathbf{u}_{c}\right|+\hat{\mathbf{d}}+\left|\mathbf{u}_{0}\right|)}{\sigma_{0}\left\|\mathbf{s}\right\|^{2}+ϒ}\right)=\mathbf{\Lambda} \end{matrix}$.

(S.45)

Then (S.44) can be written as

(S.46)

or

. (S.47)

If the matrix $\mathbf{K}_{1}-diag\left(\Lambda\right)\times diag^{-1}(\mathbf{s})$ is positive definite, then, (S.46) has the same structure as that of (S.43). Therefore, the finite-time convergence to the boundary layer is guaranteed. From (S.45) that $\mathbf{\Lambda}=\left[\begin{matrix} \Lambda_{1} & \cdots& \Lambda_{m} \end{matrix}\right]$, if the following region can be reached in finite time:

$$\left|s_{i}\right|\leq\frac{\left|\Lambda_{i}\right|}{k_{1}}$$

$$\left\|\mathbf{s}\right\|\leq\left(\begin{matrix} \left\|\mathbf{f}^{*}(\mathbf{x},\mathbf{\psi}_{f}^{*})-\hat{\mathbf{f}}(\mathbf{x},\mathbf{\psi}_{f}^{t})\right\|+\left\|\mathbf{f}_{\tau}^{*}(\mathbf{x}_{\tau},\mathbf{\psi}_{f_{\tau}}^{*})-\hat{\mathbf{f}}_{\tau}(\mathbf{x}_{\tau},\mathbf{\psi}_{f_{\tau}}^{t})\right\|+\left\|\mathbf{G}^{*}(\mathbf{x},\mathbf{\psi}_{g}^{*})-\hat{\mathbf{G}}(\mathbf{x},\mathbf{\psi}_{f_{2}}^{t})\right\|\left\|\mathbf{u}_{c}\right\|+ \\ \left\|sign(\mathbf{s}^{T})\right\|\left\|\tilde{\mathbf{\varepsilon}}_{f}(\mathbf{x})+\tilde{\mathbf{\varepsilon}}_{f_{\tau}}(\mathbf{x})+\tilde{\mathbf{d}}(\mathbf{x})+\tilde{\varepsilon}_{g}(\mathbf{x})\left|\mathbf{u}_{c}\right|\right\|+\left\|ϒsign(\mathbf{s}^{T})\right\|\left\|\frac{(\hat{\mathbf{\varepsilon}}_{f}+\hat{\mathbf{\varepsilon}}_{f_{\tau}}+\hat{\varepsilon}_{g}\left|\mathbf{u}_{c}\right|+\hat{\mathbf{d}}+\left|\mathbf{u}_{0}\right|)}{\sigma_{0}\left\|\mathbf{s}\right\|^{2}+ϒ}\right\| \end{matrix}\right)/k_{1}=\delta_{1}$$

(S.48)

For (S.47), similar to the analysis of (S.46), the following region is reached in finite time:

$$\left|s_{i}\right|^{\rho}\leq\frac{\left|\Lambda_{i}\right|}{k_{2}}$$

$$\begin{matrix} \left\|\mathbf{s}\right\|\leq\left(\left(\begin{matrix} \left\|\mathbf{f}^{*}(\mathbf{x},\mathbf{\psi}_{f}^{*})-\hat{\mathbf{f}}(\mathbf{x},\mathbf{\psi}_{f}^{t})\right\|+\left\|\mathbf{f}_{\tau}^{*}(\mathbf{x}_{\tau},\mathbf{\psi}_{f_{\tau}}^{*})-\hat{\mathbf{f}}_{\tau}(\mathbf{x}_{\tau},\mathbf{\psi}_{f_{\tau}}^{t})\right\|+\left\|\mathbf{G}^{*}(\mathbf{x},\mathbf{\psi}_{g}^{*})-\hat{\mathbf{G}}(\mathbf{x},\mathbf{\psi}_{f_{2}}^{t})\right\|\left\|\mathbf{u}_{c}\right\|+ \\ \left\|sign(\mathbf{s}^{T})\right\|\left\|\tilde{\mathbf{\varepsilon}}_{f}(\mathbf{x})+\tilde{\mathbf{\varepsilon}}_{f_{\tau}}(\mathbf{x})+\tilde{\mathbf{d}}(\mathbf{x})+\tilde{\varepsilon}_{g}(\mathbf{x})\left|\mathbf{u}_{c}\right|\right\|+\left\|ϒsign(\mathbf{s}^{T})\right\|\left\|\frac{(\hat{\mathbf{\varepsilon}}_{f}+\hat{\mathbf{\varepsilon}}_{f_{\tau}}+\hat{\varepsilon}_{g}\left|\mathbf{u}_{c}\right|+\hat{\mathbf{d}}+\left|\mathbf{u}_{0}\right|)}{\sigma_{0}\left\|\mathbf{s}\right\|^{2}+ϒ}\right\| \end{matrix}\right)/k_{2}\right)^{1/\rho} \\ \text{ }=\delta_{2}. \end{matrix}$$

(S.49)

By virtue of (S.48) and (S.49), the region $\left\|\mathbf{s}\right\|\leq\delta=\min(\delta_{1},\delta{}_{2})$ will be reached in the finite time. Since$\left\|\mathbf{s}\right\|\leq\delta$then $\left|s_{i}\right|\leq\delta$ $i=1,2,...,m$ and the terminal sliding surface (6) is written as follows:

. (S.50)

Equation (S.50) can be written as

. (S.51)

When , (S.51) is kept as the terminal sliding manifold (6). Therefore, the tracking error will converge in finite time to the following region:

$\left|e_{i}(t)\right|\leq\left(\frac{\delta}{\sigma}\right)^{\frac{1}{\eta}}$. (S.52)

This ends the proof of part 2 of Theorem 2.
